# Marginalised within a minority: Jews with disabilities in the Jewish press of the Kingdom of Poland (1860s–1914)

**DOI:** 10.1017/mdh.2023.35

**Published:** 2024-01

**Authors:** Maria Antosik-Piela, Aleksandra Oniszczuk

**Affiliations:** Faculty of History, University of Warsaw, Warsaw, Poland, Email: mm.antosik-p@uw.edu.pl

**Keywords:** people with disabilities, Jews, medicalisation, public discourse, marginalisation, Kingdom of Poland

## Abstract

This article is the first scholarly research focusing exclusively on the history of Jews with disabilities in the Kingdom of Poland from the 1860s to 1914. It analyses sources drawn from the Jewish press in Yiddish, Polish, and Hebrew. Areas of investigation include the hierarchy of attitudes towards different categories of individuals with disabilities, spiritual perspectives on disability, and the portrayal of disabilities within Jewish literature. The study places particular emphasis on the Jewish deaf community, given the proliferation of available source material. Drawing on the broad conceptual framework of disability studies, the authors examine the phenomenon of medicalisation, tracing its influence on Jewish public discourse over the latter half of the nineteenth century and the early decades of the twentieth.

## Introduction

People with disabilities are rarely seen within Polish historiography. Often relegated to the periphery of scholarly inquiry, this group has appeared predominantly either within research on special education or the activities of charitable organisations. Consequently, the exclusion experienced by people with disabilities in society finds its parallel in their continued neglect by historical narratives. This tendency is prominent when considering the various minority groups residing in the Polish lands. Despite extensive research conducted on the Jewish population, encompassing diverse historiographical perspectives, Jews with disabilities serve as a representative example of this wider trend of neglect.^1^

The limited references to Jews with disabilities within the existing historiography can be categorised as a form of ‘complementary’ history, reminiscent of early efforts to incorporate women into historical studies. Many publications describing initiatives aimed at people with disabilities in the Polish lands have such a character when they simply list Jewish organisations or benefactors.^2^ The pioneering work of Natan Meir stands out as the first historical analysis profoundly examining this subject matter. Employing an interdisciplinary approach, Meir’s study looks at three marginalised categories of individuals living in Eastern European *shtetls*: the impoverished, those with physical disabilities, and those with mental disabilities.^3^ While his scholarship is insightful, due to the broadness of the overall topic, there is still room for further research. Another noteworthy study is John Sefel’s recent research on the depiction of disability in Yiddish plays.^4^

Previous research conducted by one of the authors of this paper on different groups of people with disabilities in Warsaw—the capital city of the Kingdom of Poland—indicates that Jews experienced distinct marginalisation. The present study aims to examine in depth the social and cultural status of just this one group. A wider geographical scope is considered, as other cities of the Kingdom, which existed as a Polish state under Russian rule from 1815 to 1914, are also included. The paper examines the attitudes of the able-bodied majority—both Jewish and non-Jewish—towards the group in question. Due to the scarcity of primary sources from the early decades, the article focuses on the second half of the nineteenth century, which is more accessible due to the vigorous development of the Jewish press from the 1860s.

This study makes a number of arguments. The first is that, during this period, Jewish public discourse reflected the way people with disabilities were viewed in the wider European context; as a general rule, the way Jews with disabilities were described mirrored how their non-Jewish counterparts were perceived. Moreover, both Jewish and Polish communities founded similar aid institutions. This phenomenon can be seen as part of the process of Jewish emancipation. As the nineteenth century progressed, there was an increasing need for Jewish communities to create their own institutions, which served to both help those in need and assert their distinctiveness.

The second argument is that the marginalisation of Jews with disabilities was complex, occurring both within and outside the Jewish community. However, characterising it solely as a ‘double marginalisation’ would be inadequate because the grounds for exclusion varied, and further sub-categories can be identified within the broader category of ‘Jews with disabilities’.

The concept of intersectionality has been a valuable tool for analysis. Originating from gender studies, this concept allows social categories such as gender, ethnicity, nation, and class to be approached in their ‘interweavings’ or ‘intersections’, rather than in isolation from each other. To move beyond an ‘additive perspective’, intersectionality looks for ‘the simultaneous interaction of social inequalities’.^5^ Additionally, analytical tools originating within disability studies have been employed, in particular the social model of disability. This model views disability as a social construct distinct from the medical model, which focuses on individual physical impairments.^6^ We refer also to the concept of ‘normality’, only constructed in the mid-nineteenth century,^7^ and research on the medicalisation of disability.^8^ Discourse analysis plays a crucial role in our study as it allows us to examine the categorisations embedded in language itself.

This article investigates the perceptions and treatment of Jews with disabilities within and beyond the Jewish community, pursuing several key inquiries. These include how Jews with disabilities were perceived and treated, the presence of any hierarchical distinctions among various disabled groups, the specific considerations pertaining to Jewish women with disabilities, and the impact of broader trends such as medicalisation and societal expectations for their behaviour. Institutions providing assistance were important in shaping attitudes because of their mode of operation. Accordingly, this study asks: what kinds of institutions offered help, and what were their goals? Were the non-Jewish institutions inclusive or did they discriminate against Jews? What identity did charity and educational institutions convey? Also significant are the most difficult interactions to measure: what were the social interactions with other people with disabilities like? Were issues of nationality and ideology at stake?

This study focuses on people with vision, hearing, mobility, and mental impairments. The aggregate term ‘people with disabilities’—still non-existent in the nineteenth century—is used as a working category. An analysis is made of the statuses of various groups marginalised on the grounds of a physical or mental impairment^9^ attributed to them by society. The inclusion of deaf people within this study should not be seen as indicating the personal attitude of the authors, who share the cultural minority of the Deaf’s rejection of the label ‘people with disabilities’.^10^ We incorporate this group only due to the aggregative nature of the social discrimination against various groups of people perceived as impaired.

As is typical for most research into marginalised groups, this study had to overcome a limited availability of comprehensive primary sources and a lack of egodocuments such as memoirs or letters.^11^ Research focused on the trilingual press of the Congress Kingdom, including examples of fiction appearing in its pages. The main press titles were examined: 1) in Polish: *Jutrzenka* (1861–1863), *Izraelita* (1866–1914), *Nowa Gazeta* (1906–1918); 2) in Yiddish: *Haynt* (1908–1914), *Der Moment* (1910–1914); and 3) in Hebrew: *HaTsefira* (1862, 1874–1914) and *HaTsofe* (1903–1905). The analysis was supplemented with surviving administrative documents from the State Archives in Łódź, Radom, and Warsaw.

The Jewish population of the Kingdom of Poland made up its largest minority group and one of the largest Jewish communities in the world. An 1897 census gave a demographic breakdown of the Kingdom: mostly Catholic Poles (71.8%), Jews (13.5%), Germans (4.3%), Ukrainians (3.4%), Russians (mainly middle- and high-ranking state officials, 2.8%), and other minorities (Belorussians, Lithuanians, Armenians, and Tatars). The city of Warsaw had a Jewish population of approximately 337,000 individuals in 1914, constituting 38 per cent of total residents. This was the largest Jewish community in Europe, which has yielded researchers with an important primary source base, informing this paper’s considerable focus on Warsaw.

The Kingdom’s Jews mainly lived in towns and cities, and they were segregated into distinct groups based on religious, cultural, and political criteria. As in other Eastern European countries over the studied period, the largest group followed a traditional religious way of life and were mostly found in smaller towns, called *shtetls.* The second group, mainly residing in larger cities, identified themselves as ‘progressive’ and strove to assimilate with the Polish majority and embrace Polish cultural norms. The third group included the followers of Hasidism, an ecstatic strain of Judaism with charismatic leaders, ostensibly opposed to modernity. The fourth group consisted of the Zionists, ardent supporters of the plan to establish an autonomous state in Palestine; their ranks grew after the First Zionist Congress held in 1897. Two other populous groups included the followers of the left-wing Bund movement and the advocates of Jewish cultural autonomy within the Polish lands.^12^

This diversity of outlook was reflected in the profile of the press. Some titles aimed at integration with Polish society (*Jutrzenka*, *Izraelita*), or even assimilation (*Nowa Gazeta*), while others were interested in writing about Jewish issues ‘through a European lens’ (*HaTsofe*) or in popularising modern science (*HaTsefira* in its early years). There were also titles promoting Jewish cultural autonomy within the Polish lands (*Der Moment*) or Zionism (*HaTsefira* from the 1890s and, to some extent, *Haynt*).^13^ Despite profound differences within the Jewish population, the non-Jewish public discourse tended to perceive these groups in similar ways, displaying a range of prejudices and stereotypes about the Other, including preconceived ideas of moral degeneration and physical deformity.^14^

## Disability as a press topic

Most articles appearing in the Jewish press and mentioning disability tended to adopt an impersonal approach, rarely highlighting particular individuals with disabilities. Articles usually addressed questions of potential interest to public opinion, such as the causes or origins of specific impairments and the effectiveness of particular treatments. In line with the prevailing discourse in Poland at the time, the main subjects were deafness, blindness, and mental illness (the latter more frequently towards the end of the century).^15^ The second major topic was the lack of sufficient institutional care, regularly deplored by the Jewish press at the turn of the twentieth century.

The third general topic addressed in the Jewish press was the alleged prevalence of disabilities among Jews and likely explanations for this phenomenon. Articles eagerly relied on statistics, a prevalent tool in the nineteenth century for elucidating social and biological phenomena. The conversation surrounding this topic was not limited to Jewish titles, as the statistics cited were often reprinted from Polish or German periodicals. These analyses were based on data indicating that the proportion of Jews with disabilities was notably higher compared with non-Jews. The primary explanation put forward was the frequency of intermarriage among Jews, suggesting a correlation between consanguinity and the higher occurrence of blindness, deafness, or mental illness within the Jewish community.^16^ As for other explanations, worth recalling is the one provided by the physician Max Fischberg, who put forward that the cause of various ‘disorders’ among Jewish immigrants in New York was not in familial relationships, but rather in the historical trajectory and experiences of the Diaspora since the exile from Judea:

The greater susceptibility of Jews to nervous diseases, especially neurasthenia, hysteria, and diabetes, is the result of a long series of influences that have affected them over the past two thousand years. It is the result of constant anxiety, depression, worry, and mental overwork during medieval persecution.^17^

Fischberg’s opinion was not isolated. Some other observers identified the source of the higher percentage of Jews with disabilities (compared to Christians) with the oppression, persecution, and sanitary conditions prevalent in metropolitan Jewish districts.^18^

In the fourth type of general press article, individuals with disabilities assumed a metaphorical role, particularly in the form of epithets attributed to those perceived as irrational or fanatical. As in many European languages, the characterisation of a person as ‘deaf,’ ‘blind,’ or ‘mad’ served as a tool to critique ideological opponents, perpetuating and reinforcing the socially constructed stigmatisation of disability.^19^

Alongside these general texts, often scientific in nature, sometimes articles covering individual stories were published. These can be broadly categorised into themes related to medical, educational, professional, or poverty-related matters. Additionally, there were articles portraying individuals with disabilities as objects of curiosity. Articles focused on specific cases can be further classified based on their intention: some sought to captivate readers by highlighting extraordinary spiritual fortitude, exceptional abilities,^20^ unwavering determination in the face of adversity,^21^ or by emphasising the unpredictability of human existence.^22^ Other texts aimed to elicit compassion and encourage donations for individuals deemed ‘exceptionally miserable’ by depicting their distressing circumstances and providing personal details such as names and addresses. This approach aimed to facilitate aid by identifying those most in need of support.^23^

The very rare texts acknowledging the agency of individuals with disabilities are a separate category. Notable examples include the documented meetings of the Jewish deaf/Deaf community in Warsaw in 1911 and 1913,^24^ an article highlighting the German congress of the blind in Braunschweig in 1912,^25^ as well as references to extensive libraries for the blind in France and Vienna.^26^ Even more scarce were advertisements specifically targeting individuals with disabilities (or their families). When such announcements did appear, they predominantly catered to the deaf or blind populations. One such advertisement was issued by a teacher from the Warsaw Institute for the Deaf-Mute and Blind, Jan Sapiejewski, who claimed to treat speech impediments and develop missing sounds in both children and adults.^27^ A second advertisement promoted a Warsaw doctor who offered instruction for ‘the deaf, stutterers and people with other speech defects in clear pronunciation, writing and reading’.^28^ In a third example, the Austrian doctor of Jewish origin, Emil Fröschels, claimed to treat similar conditions. Operating a clinic in Vienna, he advertised his services in the Polish Jewish press.^29^ In another advertisement accommodation and sustenance were offered for two deaf children in the apartment of Izrael Lichtenstein, the director of an elementary school for Jewish children in Łódź, who later gained fame as a politician.^30^ A final example worth recalling is the rhyming Yiddish advertisement, which deplored the fate of the blind and promoted the use of an optician’s services:

In a world so vast, my child, beware,
When a poor man’s sight is lost in despair.
Others may possess riches and might,
But he dwells forever in eternal night.
To him, darkness becomes a divine reign,
Wealth and treasures hold no true gain.
So, my dear child, protect your precious eyes,
Preserve their strength, don’t let vision demise.
And if your sight begins to wane,
Don’t hesitate or dwell in vain.
Seek the aid that will keep your vision plenty,
Swiftly go to Nalewki 20.
^31^

## Public opinion and the hierarchy of attitudes

Public discourse during the studied period reflected varying attitudes towards individuals with disabilities, indicating that not all individuals were viewed in the same way. Rather than an explicitly recognised category, the term ‘Jews with disabilities’, as used in this study, is a working term employed to focus on individuals who were perceived by society as having a serious impairment. Common terms used in the period were the Polish word *kaleka* or the Yiddish word *kalike*, which typically translate to ‘cripple’ in English. By referring to the socially constructed idea of disability, this paper aims to highlight those individuals who had one trait in common: society believed they had a serious impairment. In most cases, it was also believed that they were deserving of both mercy and aid from the majority. Instead of a targeted analysis of just one group, this study compares and contrasts the social and cultural status of different categories of people with disabilities, revealing a hierarchy of contemporary social attitudes towards them.

Among the categories of individuals with disabilities, children with hearing and vision impairments seemed to evoke the most positive attitudes. They elicited strong compassion and, in many cases, instilled hope for future progress. Press coverage often featured deaf or blind children in the context of special institutions for their education, further emphasising the hopeful outlook associated with their potential development. Such institutions were still at a developmental stage and remained a novelty in nineteenth-century Europe,^32^ inducing a mixture of curiosity and satisfaction. The source of the latter was the chance of bringing ‘these miserable creatures’ into the world of the hearing and seeing public. Importantly, ever since the Enlightenment, a focus on children had been a major educational preoccupation—explaining the general interest in ‘improving’ those who could still be shaped freely (the *tabula rasa* concept). Consequently, it can be inferred that the positive attitude towards these children stemmed from the expectation that they would eventually shed their otherness. Similarly, adults with hearing or vision impairments were mainly depicted in a relatively favourable, or more accurately, a non-negative manner. References to these individuals frequently invoked an emotive approach, characterised by the abundant use of compassionate and pity-inducing language.^33^ People belonging to both these categories seem to be more socially accepted, although within the imposed category of ‘disability’. The deaf and the blind were still the Other, and the absence of clear negative emotions did not mean acceptance of non-standard ways of communicating, learning, or behaving.

As for people with all other physical impairments, the generic term ‘cripple’ (Yid. *kalike*, Hebr. *baal mum*, Pol. *kaleka*) was often used.^34^ These terms had an overly broad meaning, sometimes, as mentioned, referring both to the deaf and the blind. Due to the vagueness of the term, Jewish discourse surrounding ‘cripples’ lacked coherence, as the presence or absence of compassion depended on the specific context in which the term was employed. Typically, mentions of these individuals occurred within realistic or stylised narratives, or when discussing financial assistance to deserving individuals. Distinct perceptions related to people with a short stature, who were often presented as curiosities, at times eliciting stronger emotional responses. An example highlighting this sentiment was the graphic language used in a circus advertisement, which featured a ‘monstrous dwarf with the body of a six-year-old child and the head of an elderly man sporting an extravagant moustache’, dressed in children’s clothing.^35^ Presumably, this sight challenged conventional norms and standards of appearance, provoking the author of the article. It recalls the observations of Elizabeth Grosz and Natan Meir that a strong corporeal otherness, embodying deviance, both fascinated and horrified.^36^

People with mental disabilities belonged to a separate category. Traditionally, a person with mental illness or a person with intellectual disability was a familiar character within a small town or village, bearing names such as ‘village idiot’ or ‘town fool’ (Yid. *shtot-meshugener*). Some of these individuals, as described by Meir, were both sources of entertainment and objects of sympathy, integrated into the daily life of the *shtetl.* ^37^ However, migration to larger towns or cities disrupted this model. Devoid of social security, stability, and established relationships, mentally ill individuals in urban settings were no longer the familiar ‘village idiots’ but instead regarded with suspicion as unwelcome neighbours. Accounts on this issue from the first half of the century are scarce. However, we can cautiously make some inferences based on the Polish titles, which often served as a point of reference for the Jewish press. The picture emerging from these articles suggests that the public discourse in major cities largely ignored the existence of the mentally ill in the first half of the century. In contrast to the deaf, blind, or physically impaired, people with mental illnesses were noticeably absent from the lists of charitable aid recipients even during the 1860s and 1870s, both in the Jewish and Polish press. This omission may be attributed to the complex emotions evoked by the inexplicable and unpredictable behaviour associated with mental illnesses, which challenged societal control over such people. Furthermore, ideas of being possessed by malevolent spirits, or *dybbuks*, may have contributed to the perception of this group.

The medicalisation of public discourse in the latter nineteenth century brought about a shift in how individuals with mental illnesses were perceived. As in Western European states, the Kingdom witnessed an increased medical interest from the public in mental ‘disorders’, along with the wider dissemination of medical concepts. The Jewish press also began to take notice of the ’mad’, often adopting a medical standpoint, although, at times, also displaying compassion and labelling them, for example, as ‘unfortunate patients, who are unaware of their condition, for whom medical science and human mercy are trying to brighten up the gradually darkening world of thought and make it possible to communicate with their fellows again’.^38^ The medicalisation of discourse also manifested itself within the Hebrew press, particularly in articles disclosing the latest medical discoveries (e.g., the notion of inherited mental illnesses) or covering medical events (e.g., the medical exhibition held in Rome).^39^ Although some articles on mental illnesses appeared in the 1870s, their publication only became more common in the 1880s, with a fairly frequent occurrence by the 1890s and in the early twentieth century. Nevertheless, despite the emergence of medical literature on mental ‘disorders’, the strong emotions associated with this particular group persisted. From the 1890s onwards, the press fanned an atmosphere of menace, proclaiming a constant increase in the number of ‘mad’ individuals. Explanations for this trend were social and industrial changes, whereby ‘hectic, restless life brings a large group of people to a mental breakdown every year’.^40^

Despite frequent press coverage bemoaning the lack of institutions dedicated to the care of the mentally ill, personal engagement for the betterment of this group seems to have remained limited. For example, the Society for the Care of Poor, Mentally and Emotionally Afflicted Jews (Towarzystwo Opieki nad Ubogimi, Nerwowo i Umysłowo Chorymi Żydami) was an organisation that founded the ‘Zofiówka’ hospital for the mentally ill on the outskirts of Warsaw, in Otwock. As lamented in a 1912 press report:

…the Jewish population takes little interest in the society, as can be seen by the attendance at the recent general meeting held last Thursday. Of the society’s 1,216 members, only 13 showed up at the meeting. It is really sad that such a large institution enjoys so little interest among Varsovians.^41^

This might suggest that public concern for people with mental illness lacked constancy and depth, as even those who professed commitment shied away from active participation in society’s endeavours. However, this is only a cautious hypothesis, and more evidence would be needed to confirm it.

In contrast to the mentally ill, the ‘mentally retarded’ and other neuro-atypical individuals were almost completely ignored in public discourse in the Kingdom of Poland. This differentiated the state from most of the West.^42^ Throughout the nineteenth century, in many European countries people with intellectual disabilities gradually began to be distinguished from the ‘insane’ and were noticed in the public discourse. A considerable change came in the 1840s when ‘mental retardation’ began to be seen both as ‘curable’ and as a separate social problem requiring intervention. Hence, the first schools were established in France, England, and Germany. As Ido Weijers summarises,

Mental retardation became a special field of educational interest and in several countries special care for “idiots” and “imbecile” children emerged…. In the following decades social reformers, educators, and physicians developed new ideas and practical interventions towards the feebleminded. The “idiot” child was central to this new social concern…through education, the economically useless and expensive idiot could be reshaped into a useful, productive citizen who was no longer a burden to the community.^43^

Indicative of the interest in the subject were the scientific analyses published since the 1840s,^44^ the legislation affecting this group (e.g., the 1886 Idiots Act and 1913 Mental Deficiency Act in England)^45^, and the German network of institutions educating the ‘feeble-minded’ or supporting the special education system, such as the Society for the Promotion of the Education and Care of the Feeble-Minded and Idiots (Gesellschaft zur Förderung der Erziehung und Versorgung der Schwachsinnigen und Idioten)^46^.

In the Kingdom of Poland, the first medical publications focusing on the intellectually disabled appeared only at the turn of the twentieth century, ^47^ and only then did this group begin to receive some public attention. One of the reasons for the reluctance to mention it may have been the considerable social stigma associated with this group. As a Jewish author asserted in 1908, Jewish parents feared allowing their ‘mentally retarded’ children to venture outside due both to shame and the ‘fear of betraying the secret’. Instead, they confined them at home to avoid drawing attention to the child’s condition from neighbours and friends. Another reason why a child might have been kept at home was the lack of special educational institutions in the Kingdom. The first one was founded in 1904 by the Protestant (Evangelical-Augsburg) minority in Warsaw, followed in 1908 by the initiative of two Jewish women, Eugenia Lublinerowa and Dorota Zylberowa—educators, social activists, and pioneers of special education in Poland.^48^ Their school, the Warsaw Institute for the Mentally Retarded (Warszawski Zakład dla Dzieci Małozdolnych), accepted children aged 6–14 of all genders and faiths, although this openness had its limits: those with severe mental retardation (‘idiots’) were excluded, as were epileptics. The establishment of the school garnered attention from both the Jewish-Polish and Polish press.^49^

An alternative approach to addressing the issue of hierarchies of attitudes involves examining the experiences of Jewish women with disabilities. These women faced discrimination on multiple fronts due to their Jewishness, their female gender, and their disability (whether perceived as ‘deaf’, ‘blind’ ‘crippled,’ or ‘mad’). Consequently, they did not fit within one set of expectations and stereotypes. Marriage prospects for individuals with disabilities were commonly regarded as limited, and Jewish women seem to have been disproportionately affected. This conviction is poignantly expressed in the well-known book by Yekhezkel Kotik. In his memoirs, he vividly recounts his grandfather Aharon-Leyzer’s arrangements for his father’s marriage. Aharon-Leyzer insisted that the local rabbi should serve as a proxy in this endeavour:

The rabbi took the letter and showed it to Aharon-Leyzer, who was overjoyed upon reading it. “Now, my dear Rabbi, I’ll add a ruble a week to your salary,” he said, rubbing his hands together. “I intend to travel to Grodno to take a look at the girl, to make sure she’s not, God forbid, a cripple or ungainly. I love Moshe very much, so I want him to have a pretty wife, not just a girl from a good family.”

To Grodno he went, together with the rabbi. He liked the girl, and the marriage contract was drawn up…^50^

The Jewish press carried a similar attitude. In one typical story, two men, one blind and one deaf, married women whom nobody else wished to marry, as one was ugly and the other had a malicious nature.^51^ Another story concerns the plan of a Jewish man to marry off his son and echoes Kotik’s narrative:

The only thing he [my son] has to do is get married, but he has no luck… Three girls have already been proposed to me for him, and I have checked each one. And what has come out of it? One has red eyes, another has a crooked and hunched nose, another is lame. As if it were some kind of divine punishment for this poor boy…‥If he’s going to marry, he shouldn’t marry a cripple.^52^

These viewpoints reflect the marginalisation experienced by Jewish women with disabilities, regarded as ‘unfit for marriage’. For women adhering to the traditional values rooted in Judaism, with its emphasis on establishing a family and bearing children (‘a tremendous pressure to marry’),^53^ this attitude may have been particularly burdensome.

Despite the aforementioned hierarchies, a shared characteristic in the examined sources was the inferior social status assigned to all Jews with disabilities. Moreover, public opinion among so-called ‘progressive’ Jews held that individuals with disabilities possessed a further common trait: they failed to conform to the standard of utility. The general notion of utility, characteristic of nineteenth-century Europe, also permeated the Polish-Jewish press.^54^

Finally, another layer of analysis of the place of people with disabilities in the public imaginary is worth recalling. As Natan Meir has argued, the disabled—along with other Jewish marginal people—became ‘a symbol for East European Jewry as a whole’. This concerned both the external anti-Semitic narrative and the internal Jewish discourse of modernisation, progress, and integration. The latter ‘required the creation of a despised Other to serve as a kind of doppelgänger to be cast out or transformed utterly’. Thus, the disabled, as ‘all of Jewish society’s undesirable people served as scapegoats. Since in some sense they represented all of Jewish society, that society could, by sacrificing them, by placing the blame for Jewish suffering on them, redeem itself from its abject, suffering state’.^55^

## Where should Jews with disabilities go? Praise for new institutions

The tendency for social definition, categorisation, and division, increasingly seen throughout Europe during the latter half of the nineteenth century, is likewise observable within Jewish discourse. This trend is apparent when comparing emergent institutions with the traditional Jewish *hekdesh* of the early modern era. *Hekdeshim* had long served as a combination of both shelter and hospital, for locals as well as newcomers, accommodating diverse individuals including the sick, elderly, and impoverished.^56^ However, by the early nineteenth century *hekdeshim* had come to be regarded as dirty, outdated, and in disarray. Two types of institutions emerged gradually to replace them: modern hospitals designed for medical treatment and separate shelters. The latter primarily accommodated two groups: the elderly, as well as individuals with various disabilities,^57^ occasionally providing refuge for a third group—orphans.^58^ The rationale behind this collective classification was rooted in the notion that these individuals—being weak, helpless, dependent on others, vulnerable—were most deserving of public assistance.^59^

An examination of the financial records of both the Jewish and Christian shelters shows that their provision was limited to accommodation, food, and clothing. Notably, the absence of any mention of other activities undertaken within these institutions is indicative in itself, given that the press was usually eager to discuss charitable issues, even when of minor significance. We may cautiously infer that shelter residents were expected to obediently follow established rules, without exhibiting any personal interest or need. The one area where they were encouraged to display individual engagement seems to be in religious ritual.

Religion seems to have been a crucial guiding principle. Many charitable organisations and hospitals were organised according to religious affiliation, and even those of a public character were not universally egalitarian. Taking this into account, the religious identity of beneficiaries—as regarded by the institutions housing them—was especially important. For example, Jews were discriminated against at least by some Christian institutions, such as public hospitals in Warsaw that denied them assistance. This phenomenon is evidenced by the press: in 1913 the famous Jewish journalist Noah Pryłucki observed that ‘a systematic boycott of Jewish patients is now being carried out in Varsovian municipal hospitals’.^60^ Additionally, as further elaborated, Jews faced limited access to the only public school for deaf and blind children in the capital, as well as to the Society for the Deaf-Mute (Towarzystwo Głuchoniemych).

The deficiencies within institutions providing assistance to individuals with disabilities were rarely acknowledged during the studied period. Criticisms primarily revolved around the cramped facilities, exemplified by the Jewish hospital for the mentally ill, which accommodated 150 individuals despite only having 100 available spaces.^61^ Countless articles praised the idea of institutionalisation and emphasised the value of closed institutions under Jewish community leadership, such as the Main Shelter Home (Główny Dom Schronienia), for the well-being of ‘cripples’ and other dependent individuals:

Here the elderly and cripples of both sexes spend their lives free from worries and troubles, having nutritious food, warm clothing, comfortable lodging, services, medical assistance, a house of prayer, in fact everything that makes life more bearable and even pleasant.^62^

This idyllic portrayal rested on the widespread belief that institutional assistance held inherent value and that it was necessary to shield the able-bodied public from the unpleasant sight of disability, as it was unpalatable to modern aesthetics, among other factors.^63^

This trend particularly impacted individuals with mental illnesses. Once they garnered broader public attention, they became a frequently invoked group, primarily within the context of the need for new institutions. Starting from the 1890s, as modern psychiatry advanced, articles discussing the plight of the mentally ill became frequent. Common sentiment—as in the West—deemed such individuals as particularly suited for confinement within institutions.^64^ Importantly, the press asserted that the numbers of such people were increasing, fostering a sense of apprehension and perceiving them as a flood of unfortunates:

A significant group of the insane remain at large and is obviously bothering the people around. The question of what to do with all these unfortunates is becoming more and more serious, and until the conflict between the [Jewish] community and the municipality is resolved, Jewish streets will be flooded by a wave of insane homeless people.^65^

The idea of danger went so far that connotations between insanity and crime were often formulated:

Due to the small number of social institutions for the insane, the number of such people wandering around unsupervised is increasing. Insane people often go on a rampage and commit damage of all sorts. Trials conducted in these cases do not yield results, as the Napoleonic Code in force in Poland does not provide for such claims.^66^

The press often reported on arrested individuals being confined within hospitals for the mentally ill or held in police custody, further linking crime with insanity.^67^ Detention appeared to be the norm, as summarised in 1911: ‘all the lunatics that the police arrest on the streets are first taken to police custody, whence they are sent only to a hospital for the insane or to their homes.’^68^

The general view at the time deemed the mere act of confining a ‘mad’ individual within a closed facility as commendable. Confinement was seen as advantageous for both society as a whole and the individual. This brief acknowledgment in the press is emblematic of the prevailing mindset: ‘I feel it is my duty to thank the Honourable Mrs [wife of] Natan Morgenstern for the activities she developed in an effort to *alleviate the misery* of my insane employee *by placing her in an institution* for the insane [emphasis added]. I express my humble thanks for this. *S. Nuremberg.*’^69^ Press mentions of these institutions repeatedly employed language pointing at the need for segregation.^70^ Theories on hospital organisation reflected this approach, advocating for the grouping of all wards, except those for patients with infectious diseases and the ‘insane’.^71^

One prominent issue of concern for the reading public was the deservingness of public aid and the direction of institutional development. According to a Yiddish article from 1912, ‘cripples’ do not deserve the aid of public hospitals. The author lamented that ‘cripples’, along with the elderly and other incurable individuals, constituted one-fifth of all patients in Varsovian hospitals, thus ‘taking the place of other’ ill individuals.^72^

A different face of the issue of ‘undeservingness’ was the segregation of people perceived as disabled on the basis of religion or nationality, which affected deaf/Deaf Jews in Warsaw at the turn of the twentieth century.

## Among the deaf and blind in Warsaw: equality, exclusion, empowerment

The Warsaw Institute for the Deaf-Mute and Blind occupied a prominent position in the Jewish public sphere, particularly among those seeking greater integration with the ethnic Poles. This establishment has left a substantial collection of primary sources allowing a closer examination of a specific group of Jews perceived as impaired. Despite being established by a Catholic priest and predominantly managed by clergymen, the Institute accepted boys and girls of all faiths for several decades.^73^ Jewish pupils typically constituted a small percentage of the overall student body.^74^ Since its founding in 1817, the institution garnered praise from both Polish and, subsequently, Polish-Jewish public opinion, viewed as a wholly beneficial endeavour and a testament to new medical and educational advancements. By focusing exclusively on children, the Institute aligned with the Enlightenment’s emphasis on educating the young, who were seen as malleable and holding future promise. The financial contributions made by Warsaw’s elites to sustain the Institute gained popularity, with donors eagerly offering their support and, importantly, sharing information about their philanthropy with the press. The so-called progressive members of the Jewish community, seeking social and cultural integration with the Polish elite, were also eager to contribute public donations to the Institute. This charitable activity not only provided an opportunity to assist deaf and blind children but also served as a means for benefactors to gain publicity for their generosity in supporting non-Jewish philanthropic institutions.^75^ For instance, in 1873, when plans to construct the second floor of the Institute’s building were announced, the Jewish press promptly informed its readers: ‘Our wealthier co-religionists generously contribute to create the funds needed for this enterprise.’ A few months later, the same press hailed Lesser Lewi, ‘the banker, the Jewish community board member, and the commercial counsellor’ for having ‘donated the entire missing amount to complete the construction’.^76^ At that time, some members of the Jewish community considered this matter more pressing than the construction of a synagogue.^77^ In this manner, benefactors advanced the agenda of the ‘civilised’ or ‘Enlightened’ Jewry, seeking integration. A similar trend could be observed in other European countries, as wealthy Jews made contributions to Christian institutions or mixed institutions to demonstrate their openness.^78^ Interestingly, the story of an 1818 bequest allegedly designated by Berek Sonnenberg, the wealthiest Jew in the Kingdom of Poland, also served this purpose. As Marcin Wodziński has found, pressure from state authorities prompted Berek’s heirs to ‘clarify’ his will and donate a substantial sum to the Institute.^79^ Throughout the century, the story of Berek’s contribution remained well-known, presumably inspiring other wealthy Jewish donors to follow suit.^80^

Until at least the mid-1880s, there existed a relatively balanced relationship between the financial contributions made by Jewish individuals and the Institute’s openness toward Jewish children. Any mention of the Institute in the Jewish press was consistently positive, with Jan Papłoński, the director from 1867 to 1885, being particularly admired. Papłoński exhibited an open attitude toward Jewish pupils. For instance, in 1873, he announced the creation of two additional scholarships from the Institute’s funds, alongside an existing scholarship for a Jewish child, allowing three children of the Jewish faith to study at the Institute free of charge.^81^ Under Papłoński’s leadership, outstanding Jewish students of the Institute were eligible for annual prizes awarded for ‘distinguished diligence, progress in studies and exemplary performance’.^82^ This not only recognised the behaviour and achievements of these pupils, but also the attitude of school authorities who held up these pupils as role-models for other children. Notably, Papłoński repeatedly called for a ‘teacher of religion’ for the Jewish students, emphasising equality in education.^83^ Equality was further manifested in the founding documents of the Association of the Deaf and the Association of the Blind, both established in 1883 on Papłoński’s initiative.^84^ Similarly, the Tavern at Piwna Street in Warsaw welcomed all deaf and blind individuals, irrespective of their religious or social backgrounds.^85^

The experience of Jewish students at the institute was not only conditioned by the director’s approach, but also by the everyday interactions between students and teachers. Due to the absence of personal accounts, our understanding relies on external observations. There were several mentions in *Izraelita* praising the camaraderie among pupils in the 1870s and early 1880s, including this excerpt:

What seized the heart was the handing out of awards for camaraderie, which were awarded by a majority of votes by the students themselves. Unspeakable joy beamed from the eyes of the chosen, and tears of happiness muffled their voices. We were all the more moved by the fact that among those chosen and awarded for camaraderie were three Jewish pupils. This proves, among other things, the spirit of tolerance and feelings of brotherhood, which the venerable head of the institution and his esteemed colleagues are trying to instil in the pupils.^86^

Following Jan Papłoński’s death in 1885, this emphasis on equality began to wane. The first catalyst for change was the policy of Russification implemented in the Institute from 1886 until the outbreak of the First World War. As part of this policy, only Russians were appointed as directors of the Institute, reflecting the Russian Empire’s desire to exert control over educational institutions and influence the minds of the young. Accordingly, in 1886 a separate Russian curriculum was introduced at the Institute. A few years later, fifty-nine Orthodox children were enrolled in this course, in addition to eleven Protestants and fourteen Jews.^87^ A similar trend occurred in the education of the blind, where Russian became the language of instruction for all students, despite only a small number of Orthodox Christians being present. Only additional education in Polish was allowed.^88^ We may assume that this policy signified a shift away from the interests of some Jewish parents, for whom Polish acculturation held greater appeal than the Russian language and culture. Integration for the Kingdom’s Jewish population was synonymous with Polish acculturation, as evidenced by historical research. The Russian language and culture held less appeal in this context.^89^ The second unfavourable development was the implementation of limits on the admission of Jewish children.^90^ From at least 1904, a departure from previous practices took place, allowing only Christians to attend the school as beneficiaries of public funding. Jewish pupils were treated similarly to foreigners and could only be accepted as ‘private students’,^91^ thereby impeding access for poorer Jewish children. Additionally, percentage quotas further restricted the enrolment of Jews. Unsurprisingly, the idea of establishing a separate school gained traction among deaf Jews in Warsaw.^92^

In addition to the organisational changes discussed above, there may have been another factor undermining Jewish interest in the Institute. A press account from 1913 sheds light on the relationship between Jewish and non-Jewish deaf individuals, revealing a far from neutral dynamic. A hearing journalist who attended a gathering of Jewish deaf/Deaf in Warsaw, provided the following summary of the situation:

The deaf all over the world, as we know, have their own schools where they learn to read and write, their own associations that provide them with material and spiritual support, and also in Warsaw, where more than 1,000 deaf people live, there is a society with some capital and a special school where these unfortunates learn the “language” and have the opportunity to organise themselves through this. It would seem that there is a real unity among these unfortunates, that misfortune will make of them one organised group….However, also among the deaf, anti-Semitism has broken in, also here the language issue has arisen, also in this group there are fierce nationalists, chauvinists, boycotters, etc., just like among the hearing.^93^

This disagreement between the two groups indicates their susceptibility to external influences and ideologies, with anti-Semitism being a growing issue.^94^ An exclusionary policy of the time further hindered the access of poorer Jews to the sole institution that facilitated social interactions and empowered the Warsaw deaf/Deaf community:

The charter of the Warsaw Association of the Deaf clearly states that any deaf person, regardless of nationality, can belong to the association, but this paragraph only functions on paper, and in reality, a “percentage quota” for Jews began to be introduced, and among the Jewish deaf, only a few, those more affluent and more educated, were accepted.^95^

This 1913 gathering, aimed at establishing a Jewish association for the deaf/Deaf, was not the first indication of a desire for separation within this group. In 1911, a similar meeting had taken place, gathering 200 Jewish men and women with the objective of establishing an institution ‘whose task would be to bring together the social life of deaf Jews’.^96^ This initiative was realised in 1916.^97^

Indirect evidence of segregation between Jewish and Christian deaf individuals was also to be found outside Warsaw. An 1898 administrative poll conducted in the industrial city of Łódź suggests that some deaf residents from the same building may have formed into groups. Based on age distribution, it may cautiously be inferred that these clusters did not consist of family members but rather of individuals who voluntarily gathered in friendship groups.^98^ Notably, there were no cases of Jewish and Christian deaf individuals living at the same address. In a few cases, such ‘enclaves’ comprised Christian alumni of the Warsaw Institute for the Deaf-Mute and Blind, with a total of sixteen recorded alumni in Łódź. It is noteworthy that, no Jewish alumni from the Institute resided in Łódź during that time.^99^ The absence of mixed communities may suggest this was a moment when nationality played an important role among the deaf. Moreover, Jewish and Christian deaf individuals outside Warsaw, where a joint school for the deaf existed, may simply have lacked a common language of communication. It is likely that most Jews who relied on oral language and lip-reading would have known only Yiddish, while those who used sign language would have used home signs developed separately within each family.

The increasing prominence of national identity among the deaf/Deaf community might have paved the way for the establishment of two separate schools for Jewish children. The first Jewish school for deaf children was founded by Litman Himelsztajn in the small eastern town of Międzyrzec Podlaski in 1901, followed by the Jewish School for Deaf Children established by the Jewish Society for Deaf Assistance, *Esras Ilmim,* in Łódź in 1910, directed by the aforementioned Izrael Lichtenstein.^100^ Compared to neighbouring regions, this phenomenon emerged relatively late.^101^ These endeavours were part of a trend of establishing separate institutions for Christians and Jews throughout the Kingdom of Poland. While members of both groups had formerly participated in shared philanthropic associations throughout most of the nineteenth century, its latter decades witnessed a visible increase in the number of independent Jewish institutions in numerous cities within the Kingdom^102^.

## Spiritual approach: disability and folklore

Medicalisation was not the sole factor impacting the status of individuals with disabilities within the Jewish community. Both the spiritual heritage of Judaism as well as folk beliefs played a further role. The latest research indicates that Judaism did not exhibit a consistent attitude towards disability due to its foundation on discussion and the allowance of diverging opinions. Despite this, Judaism could serve as a basis for the marginalisation of people with disabilities, as it included beliefs that associated disability with sin and saw suffering as an act of atonement.^103^

Folk beliefs, which have received less scholarly attention, played a meaningful role, particularly the ‘Hasidic superstitions surrounding the “evil” inherent in people with disability’. Themes related to spirituality were also present in the stories of Hasidic leaders (*tzaddikim*), performing miracles of healing.^104^ Although the Polish-Jewish press, especially *Izraelita*, vehemently opposed Hasidism and all sorts of pre-modern concepts, occasional articles on Jewish folklore and folk medicine were published.^105^ These writings included advice and recommendations on medical prevention and treatment, some of which aimed to protect Jews from physical and mental impairments. Many of the superstitions related to disability were associated with the mother’s behaviour during pregnancy. For instance: ‘When the mother looks at ugly things, cripples, or animals, the child is born a monster.’^106^ Folk belief attributed mental illness to practices such as inadequate hand washing or passing through an area where nail clippings were discarded, as nails were believed to possess an ‘impure force’ capable of causing madness.^107^ Folk belief also emphasised the ‘correct’ behaviour towards people with disabilities: ‘One should also not imitate lunatics, for the result is that one can easily go crazy. One should never mock and mimic cripples, for it is easy to become crippled by this.’^108^ Various rituals existed, including those purported to cure epileptic patients, some involving the use of animals such as tearing a live black hen over the sick person’s head or applying mole ointment. Another practice involved decapitating a rooster and burying its head beneath a barn.^109^ These customs were not exclusive to Jewish folklore and had similarities with Polish traditions.^110^

Additionally, cholera weddings, also known as black weddings or *Shvartze khasene* in Yiddish, were held during the nineteenth and early twentieth centuries.^111^ Natan Meir describes this ritual as one of the most distinct practices within ‘the rich spiritual matrix of East European Jewry’.^112^ During cholera epidemics, the Jewish community organised cemetery weddings for poor orphans, including those with disabilities.^113^ This ritual aimed to safeguard the town’s community from contracting cholera, as the organisers believed that the intercession of deceased ancestors would invoke God’s mercy and protect people from illness and death. As aptly noted by Meir, it was a ‘corrective ritual intended both to normalize marginal people through marriage in order to achieve a kind of cosmic reconciliation that would banish the epidemic, and to remove the calamity from the mainstream of the Jewish community to the community’s chosen scapegoats: its marginals’.^114^

Reports of cholera weddings in the Kingdom of Poland appeared intermittently in both the Polish and Jewish press, as for example this event organised in 1894 in the town of Skierniewice:

A thousand Jews on foot, on horseback or on carts, proceeded in one direction, with music at the forefront. The pedestrians participating in this strange procession, with their body movements resembled some kind of wild choreography, and the entire procession was preceded by Jews dressed in red pantaloons with yellow side stripes, with bizarre hats on their heads.^115^

The assimilated Jews of Warsaw held an ambiguous attitude towards the peculiar custom of cholera weddings. On the one hand, these events were viewed as superstitions unrelated to Judaism, while on the other hand, they were seen as innocent beliefs that brought relief and solace to people^116^ and which writers in *Izraelita* did not wish to condemn. This press title also reprinted an article from a local Polish daily, *Gazeta Lubelska*, on a wedding held in a Lublin cemetery featuring a couple of ‘deaf-mute paupers’.^117^ The editors of the Polish publication considered the superstition harmless. In the context of weddings involving individuals with disabilities, the Yiddish Warsaw daily *Der Moment* reported on the marriage of a Jewish couple from Warsaw:

Yesterday, on Pokorna Street, a wedding took place between a well-known local idiot and a poor, homeless orphan, who is also not very sane. The groom’s parents are known throughout Warsaw by the names ‘Hershele’ and ‘Sorele’ and wander the streets constantly. This unusual wedding attracted hundreds of onlookers who were willing to pay just to be invited to the wedding.^118^

These customs coexisted with the nineteenth-century processes of rationalisation and modernisation, influencing attitudes towards disability. Occasionally, they faced criticism from a medical standpoint, suggesting that the union of two mentally ill individuals would transmit these diseases to future generations: ‘The reader of *Der Moment* certainly read about this kind of wedding in one of our issues a few days ago. In this case, both the bride and groom and the parents were insane. It goes without saying how dangerous such a ceremony is. This is how the next generation of idiots and insane grows.’^119^

These superstitions fostered the belief that the aetiology of disability possessed a spiritual nature, leading to the perception that individuals with disabilities required supernatural treatments rather than medical ones. Importantly, these ideas reinforced the perception of otherness within the analysed group.

## Disability in fiction

Disability also found its place in nineteenth-century Jewish literature, such as Mendele Moycher Sforim’s novel *Fishke der krumer* (Fishke the Lame, 1869)^120^ and Meir Weissenberg’s short story *Di meshugene in dorf* (The Village Madwoman, 1905).^121^ Although literary works were occasionally published in the Jewish press, those featuring characters with disabilities were relatively few in number. Lennard Davis, who has examined the portrayal of people with disabilities in European novels, observes that ‘if disability appears in a novel, it is rarely centrally represented. It is unusual for a main character to be a person with disabilities.’^122^ The so-called ‘village idiot’, an integral part of the *shtetl* landscape mentioned above, was a stock character in Jewish fiction. Typically depicted in a supporting role, they were always described in relation to their interactions with the local community.^123^ Despite some inhabitants expressing sympathy, their presence was primarily marked by disregard. Mentally ill characters were often treated as objects of amusement and subjected to jokes and challenges, particularly from children:

Leaning against the fence stands poor Trajna, unconscious; tears are pouring from her dull eyes.…

A bunch of urchins have arrived, tearing her clothes and shawl.

- Be off from here, naughty children! Be off from here!

- Trajna madwoman! Trajna madwoman!

And she cries…then laughs again!^124^

Henryk Lichtenbaum, editor of *Izraelita*, expressed his sentiments similarly. While visiting the industrial town of Zgierz, he encountered a mentally ill man who evoked childhood fears of a similar ‘village idiot’, leaving Lichtenbaum profoundly horrified and shocked.^125^ The historical novel *Potwarca* (The Slanderer) features the mentally ill character of Carrabas, an object of ridicule by the local community:

The appearance of Carrabas in the town was sometimes a source of great delight for the street folk. His infirmity, his twitching coldness, and the perpetual smile on his lips, provoked their taunts, which the cripple endured not only good-naturedly, but even with apparent satisfaction. Whole groups of street youths chased after him, tugging at him on all sides, throwing mud at him and teasing him in every possible way, and they could never make him angry.^126^

The same pattern of relations between the mentally ill and local children appears in the story *Sane der kapelmeister* (Sane the Bandmaster). Here too, the protagonist—who ‘assumed the function of the village idiot’—is a ‘source of amusement and ridicule’^127^ whom the children chase. However, in this case, the hero beats the boys who tease him, which causes outrage in the *shtetl.* In literary works published in the Jewish press, mentally ill individuals were depicted in a similarly schematic manner, aligning with Natan Meir’s observation that ‘town fools’ were regarded as ‘figures of fun and sources of entertainment’.^128^ However, character descriptions were rarely in-depth, and authors typically focused solely on painting disabled characters as separate and distinct from the other *shtetl* inhabitants.

Another group of protagonists in these literary works were individuals who were blind, deaf, or physically disabled. However, such figures were scarce in the texts published in the Jewish press, and they generally did not assume leading roles. Such texts often aimed at highlighting their physical appearances. For instance, in a story by Czesława Endelmanowa, a hunchbacked character named Josel is portrayed as resembling a ‘large monstrous spider’.^129^ In a play by Jan Adolf Hertz, a blind father is grateful to his son for taking care of him while simultaneously experiencing sorrow for never having seen the face of his beloved child. ^130^ The press also published works that addressed the deafness of soldiers, as seen in *Przygody głuchego weterana* (Adventures of a Deaf Veteran)^131^ or *Przygody siłacza* (Adventures of a Strongman),^132^ where stories of war invalids are described.

However, both in these works and in texts featuring ‘village idiots,’ the presence of disabled characters primarily serves to contrast them with protagonists who are not disabled. They were portrayed as unfortunate individuals eliciting pity, with their disabilities repeatedly emphasised. While they are part of the local community, they do not function on equal terms with other residents. Instead, as dependents of their families or the Jewish community (*kehillah*), they occupy a marginal position within the daily life of the *shtetl.*

## Conclusions

Jewish public discussion of disability in the second half of the nineteenth century largely echoed the prevailing non-Jewish discourse. This was particularly evident for the Polish-Jewish press, where so-called ‘progressive’ members of the Jewish community sought integration with Polish society and acculturation into Polish culture. Two periodicals, *Jutrzenka* and *Izraelita*, explicitly followed this path, employing the rhetoric of pathos and repeatedly hailing the concepts of progress, enlightenment, and civilisation. The third Polish-Jewish title, the assimilationist *Nowa Gazeta*, took a similar approach. But even the Yiddish and Hebrew titles were not isolated from the wider European discourses on disability and medicine. The journalists of *HaTsefira*, particularly in the first three decades of its existence, aimed to popularise modern technology and science, especially the natural sciences. Even as this periodical evolved towards Zionism, minimising the importance of popularising science, the change did not impact the attitude towards people perceived as disabled. Similarly, *Der Moment* and *HaZofe*, despite concentrating on Jewish topics, did not present any specific approach towards the group in question.

The grounds for the transfer of ideas into the Jewish press were manifold. First, it was the bilingualism or multilingualism of its writers,^133^ which gave them access to the non-Jewish discussions about medicine and disability. Second, the medicalised discourse on disability was widespread in public institutions such as hospitals and shelters, and this had an impact on the way disability was understood and discussed. Third, the ubiquity of medical discourse in the Jewish press can also be attributed to the high number of Jewish doctors and their involvement in medical societies. They were part of the socially engaged intelligentsia that led social campaigns to bring about progress by improving the health and hygiene standards of the population. Since the 1860s, when Jews were granted full citizenship in the Kingdom, these institutions had an inclusive character and accepted Jewish individuals. As Aneta Bołdyrew summarises, ‘the medical societies enjoyed the best operating conditions of all the scientific organisations. They were somewhat less subject to censorship, enjoyed greater freedom of assembly than other intellectual circles, and brought together well-educated specialists.’^134^ Some Jewish doctors had acquired experience not only through participation in international medical congresses, but also through studies, study tours, or work abroad, particularly in Germany, Austria, or France, and subsequently introduced Western medical concepts and approaches to the Kingdom of Poland. For instance, Stanisław Leopold Lubliner (1863–1937), a specialist in laryngology and pulmonology in two Warsaw hospitals (and husband of the aforementioned Eugenia Lublinerowa), after completing his studies, visited two clinics in Berlin and four clinics in Vienna. Following his stay in clinics in Davos and Hamburg, he introduced the use of artificial emphysema in Warsaw. Lubliner was also co-owner and co-editor of the periodical *Kronika Lekarska* (Medical Chronicle).^135^ Another example is Wilhelm Szymon Lubelski (1832–1891), a psychiatrist from Warsaw who had practised in Paris and Vienna, contributed to the Warsaw Medical Society (*Towarzystwo Lekarskie Warszawskie*), and was involved in organising its psychiatric section. He also authored numerous articles for Polish medical journals.^136^

One additional factor impacting the perception of disability was the omnipresent discourse of utility that strengthened the division of society into two groups: the self-reliant and those perceived as a public burden. This societal division was influenced by industrialisation, which introduced new ideas of efficient labour based on time and motion.^137^ This resulted in the inferior classification of individuals deemed neither independent nor able-bodied.

Despite the widespread practice of ‘othering’ Jews with disabilities and perceiving them as inferior, varying attitudes towards individuals with specific disabilities existed within a hierarchical framework. It is worth exploring why these attitudes were diverse and why certain groups were viewed more positively (or rather, less negatively) than others. One explanation for this hierarchy of attitudes lies in the social policy of the Kingdom of Poland. By placing particular emphasis on the education of the deaf and blind, as evidenced by the establishment and continuous funding of the Warsaw Institute for the Deaf-Mute and Blind (and similar institutions were planned in other cities),^138^ state authorities directed public attention towards this specific group of individuals. The Institute served as a model institution, attracting great interest and support from both Polish and Jewish financial elites, who willingly made donations and participated in annual open examination sessions. The media also took notice of the Institute, praising its work. Since it was the sole school for children viewed as disabled for a large part of the century, the education of the deaf and blind was regarded as a distinctive and captivating endeavour. It was also believed that such an educational effort would minimise the undesirable social separation and otherness of these children and that their vocational training would make them socially useful.

Another factor influencing the varied perception of disability in the period under study was the content of medical brochures and books. Doctors believed in the importance of popularising medical knowledge, and a large number of publications in the Kingdom of Poland focused on two impairments: visual and auditory disabilities. What might have made the subject of deafness particularly popular were the medical theories that promised successful therapies. There were many accounts of the latest medical and pedagogical tools aimed at treating hearing loss and overcoming communication barriers.^139^ Towards the end of the century, with the growing Western interest in mental illness, the Jewish public in the Kingdom could read more about another group of people perceived as disabled. Frequent press stories mentioning mentally ill individuals mirrored the broader perception of those people as dangerous and uncontrollable, necessitating confinement in psychiatric hospitals. Constant reference to the increasing size of the ‘insane’ population and limited capacities of existing institutions fuelled a sense of threat.^140^

Strong emotions, although of another kind, also impacted the place of neuro-atypical people in the public imaginary. As a rule, they seem to have induced shame in their relatives and thus were mostly kept at home. Until the early 1900s, the analysed press was silent about this category of individuals, reflecting the general lack of medical publications or educational institutions aimed at this group in the Kingdom of Poland. Due to this silence, we may speak of a clear distinction between neuro-atypical people and other individuals with disabilities, particularly the deaf and the blind.

In addition to broader trends, a specific Jewish approach played a role in shaping attitudes towards disability. Recent research by John Michael Sefel highlights the significance of the core ideas of Haskalah, the Jewish Enlightenment born in the late eighteenth century. Its supporters, known as Maskilim, ‘sought a transformation of Jewish life and culture through major reforms to religious, educational, and social standards. Through championing the cultural and scientific advances already being embraced by Western bourgeois society, the Maskilim hoped to ultimately bring about – as described by Shmuel Feiner – a “phenomenon of transition from tradition to modernity”.’^141^ In the context of the current study, worth stressing is the ‘maskilic view of the causation between anti-modernisation and disability, of anti-science and disease’,^142^ a perspective evident not only in publications like *Izraelita*, which represents late maskilic ideology, but also to some extent in other analysed periodicals. A similarly unfavourable setting for disability was provided by Zionist concepts, present in *HaTsefira* (from the late 1890s) and to some extent *Haynt.* They heralded the creation of the ‘New Jew’ and rejected the image of its opposite in the form of a weak Diaspora Jew. In this regard, Zionism

…harboured profound ambivalence about the disabled Jewish body and psyche: they represented the persecuted, wounded, and deeply unhealthy Jewish nation, and therefore merited concern and sympathy. At the same time, however, they were also the clearest example of the Jews’ degenerate state and therefore had to be done away with through the regeneration and transformation that Zionism promised.^143^

Taking the broader perspective suggested by Natan Meir, with the advent of modernity, as ‘is almost universally the case of social outcasts’, people with disabilities and other marginals began ‘to play a fraught role as the symbolic Other for Jewish society, which projected onto them its anxieties about its perception in the eyes of the Christian world, the rapid impoverishment of … Polish Jewry’. Importantly, ‘Modernizing reformers and philanthropists were particularly concerned that large groups of marginal types, more visible to Christian society in the cities to which Jews were migrating in large numbers, might endanger the positive image of modern Jewry that progressives were attempting to cultivate.’^144^ Facing external stereotypes and concepts on Jewish alleged moral degeneration and physical deformation, the Jews displaced these features ‘onto their destitute and disabled’.^145^

The Jewish press played a key role in perpetuating and diversifying attitudes towards people with disabilities, with a common thread being their treatment as the Other. The marginalisation experienced by individuals with disabilities could manifest in varying forms depending on circumstance, such as discrimination based on Jewishness, gender, deafness, blindness, physical impairment, mental illness, poverty, or as beneficiaries of public assistance. These marginalising attitudes, famously described by Harlan Lane, were disguised behind a ‘mask of benevolence’ maintained by the ‘able-bodied’.^146^ Interestingly, the attitudes of the ‘able-bodied’ also influenced those of people with disabilities. The prominence of the national question among the deaf/Deaf community in Warsaw at the turn of the twentieth century serves as evidence of intersectional labelling within the disabled community.

Furthermore, as the nineteenth century witnessed a growing interest in the human body, individuals with physical impairments in the Polish lands began to be perceived through this lens. Their bodies were ‘interpreted through cultural values and expectations surrounding physical and mental ability’.^147^ The exotic bodies and rituals of non-European people were eagerly observed by both Polish and Jewish audiences, becoming ‘a democratised form of mass entertainment’.^148^ Paradoxically, the disabled body – considered at odds with the concept of normalcy – was expected to remain hidden, preferably within the newly established ‘progressive’ institutions established to provide for them.

## Footnotes

1 For a general discussion on Polish historiography, see: Moshe Rosman , ‘How Polish is Polish history? Polish history’s problem of definition,’ in Yvonne Kleinmann et al. (eds), Imaginations and Configurations of Polish Society: From the Middle Ages through the 20th Century (Göttingen: Wallstein Verlag, 2017), 19–36; Patrice Dabrowski , ‘On Forgetting, Displacement, and Historical Error in Polish History’, The Polish Review, 66, 4 (2021), 6–12 .

2 See for instance: Elżbieta Mazur, *Dobroczynność w Warszawie XIX wieku* (Warszawa: Instytut Archeologii i Etnologii PAN, 1999); Kazimierz Badziak and Jacek Walicki, *Żydowskie organizacje społeczne w Łodzi (do 1939 r.)* (Łódź: Wydawnictwo Ibidem, 2002); Marek Przeniosło (ed.), *Dobroczynność i pomoc społeczna na ziemiach polskich w XIX, XX i na początku XXI wieku*, vols 1–2 (Kielce: Kieleckie Towarzystwo Naukowe, 2008–2010); Beata Trębicka-Postrzygacz , Szkoły dla dzieci niesłyszących na ziemiach polskich w latach 1817–1914 (Lublin: Towarzystwo Naukowe KUL, 2011); Mirosław Łapot , ‘Działalność szkół i zakładów dla dzieci niemych i niewidomych, założonych z inicjatywy Żydów lwowskich w latach 1871–1939’, Szkoła Specjalna, 4 (2018), 259–71.

3

Natan M. Meir , Stepchildren of the Shtetl: The Destitute, Disabled, and Mad of Jewish Eastern Europe, 1800–1939 (Stanford: Stanford University Press, 2020).

4

John M. Sefel , Staging The [Disabled] Jew: The Thematic Use of Doctors, Disability, and Disease in Yiddish Plays on Modernization, 1790–1929 (PhD Dissertation, Ohio State University, 2021).

5 Katharina Walgenbach, ‘Intersektionalität als Analyseperspektive heterogener Stadträume,’ in Elli Scambor and Fränk Zimmer (eds), *Die intersektionelle Stadt. Geschlechterforschung und Medien an den Achsen der Ungleichheit* (Bielefeld: transcript Verlag, 2012), 81. For references to this concept within disability studies, see: Alison Kafer and Eunjung Kim , ‘Disability at the edges of intersectionality,’ in Clare Barker and Stuart Murray (eds), The Cambridge Companion to Literature and Disability (Cambridge: Cambridge University Press, 2017), 123–38; Heike Raab, ‘Intersektionalität und Behinderung – Perspektiven der Disability Studies‘, *Portal Intersektionalität* (2012).

6

Tom Shakespeare , ‘The social model of disability’, in Lennard J. Davis (ed.), The Disability Studies Reader (New York: Taylor and Francis, 2006), 214–21.

7

Lennard J. Davis , ‘Constructing normalcy. The bell curve, the novel, and the invention of the disabled body in the nineteenth century’, in Lennard J. Davis (ed.), The Disability Studies Reader (New York: Taylor and Francis, 2006), 3–16 .

8

Peter Conrad , The Medicalization of Society: On the Transformation of Human Conditions into Treatable Disorders (Baltimore, MD: The Johns Hopkins University Press, 2007).

9 Academic terminology relating to categories of people with disabilities is not standardised. Among the most problematic and vague are terms relating to ‘mental disability’. In the current study, this term includes two groups: people with ‘mental illness’ and people with ‘intellectual disability’ (‘mental retardation’). For a discussion on terminology, see: Gillian Ray-Barruel, ‘The Legacy of Special Education in Victorian England’, *The British World: Religion, Memory, Culture and Society conference.* *Conference Paper* (July 2012), 389–403; Ido Weijers , ‘Educational Initiatives in Mental Retardation in Nineteenth-Century Holland’, History of Education Quarterly, 40, 4 (2000), 461 .

10

Cf. Don Miller , Damned for Their Difference: The Cultural Construction of Deaf People as Disabled: A Sociological History (Washington, DC: Gallaudet University Press, 2002); M. Zaurov, *Gehörlose Juden: Eine doppelte kulturelle Minderheit* (Frankfurt am Main: Peter Lang, 2003).

11 One of the very few voices of people with disabilities, although with the intermediary of the press: ‘A kleyne bakoshe’, *Haynt*, 99 (12 May 1912), 5; M. D., ‘A ferzamlung fun toyb-shtime’, *Haynt*, 38 (26 February 1913), 4–5.

12 The scholarly literature on the diversity of Polish Jewry in the nineteenth century is vast; see for example: Antony Polonsky , The Jews in Poland and Russia: Volume II: 1881 to 1914 (Oxford: Littman Library of Jewish Civilization, 2010); Marcin Wodziński , Haskalah and Hasidism in the Kingdom of Poland: A History of Conflict (Oxford: Littman Library of Jewish Civilization, 2005); cf. also: Ezra Mendelsohn , On Modern Jewish Politics (Oxford: Oxford University Press, 1993).

13 For more on these press titles: Marian Fuks, *Prasa żydowska w Warszawie 1823–1939* (Warszawa: PWN, 1979); Joanna Nalewajko-Kulikov (ed.), *Studia z dziejów trójjęzycznej prasy żydowskiej na ziemiach polskich (XIX-XX w.)* (Warszawa: Wydawnictwo Neriton, 2012).

14 See e.g.: Theodore T. Weeks , ‘Jews and Poles, 1860–1914: Assimilation, emancipation, antisemitism’, in François Guesnet et al. (eds), Polin: Studies in Polish Jewry, 31 (Oxford: Littman Library of Jewish Civilization), 121–42; Joanna Michlic , Poland’s Threatening Other. The Image of the Jew from 1880 to the Present (Lincoln: University of Nebraska Press, 2006); Natan M. Meir, *Stepchildren of the Shtetl*, *op. cit.* (note 3), 83.

15 See e.g., ‘Yediyot ha-olam ve-ha-teva. Holey ha-guf ve-holey ha-nefesh’, *HaTsefira*, 28 (26 July 1876), 4–5; ‘Zichronot me-asfat ha-rofim be-Romi’, *HaTsefira*, 95 (11 May 1894), 3; N. L., ‘Visenshaftlikher felyeton. Toybe zolen heren’, *Haynt*, 127 (15 June 1914), 3.

16 See: ‘Przegląd pism krajowych’, *Jutrzenka*, 39 (24 September 1862), 319; ‘Małżeństwa między krewnymi’, *Izraelita*, 37 (18 September 1891), 352; ‘Śmiertelność i choroby u żydów i nieżydów’, *Izraelita*, 39 (23 September 1904), 455; ‘Małżeństwa między krewnymi’, *Izraelita*, 4 (24 January 1908), 36; ‘In der yudisher velt. Di likvidatsye fun der shkhite-frage. Golus un meshugas’, *Der Moment*, 110 (24 May 1914), 2. Considerations in the Jewish press were part of a broader perspective on intermarriage. These issues have also been analysed in the Polish press, see e.g., Jan Papłoński, ‘Skutki związków małżeńskich pomiędzy osobami spokrewnionymi’, *Kurjer Warszawski,* 258 (20 November 1868) 2; Jan Papłoński, ‘Wpływ małżeństw między osobami pokrewnemi na głuchoniemotę i inne kalectwa’, *Pamiętnik Warszawskiego Instytutu Głuchoniemych i Ociemniałych* (Warszawa, 1871), 56–8; Marceli Brzeski, ‘Dwanaście instytutów głuchoniemych’, *Pamiętnik Warszawskiego Instytutu Głuchoniemych i Ociemniałych* (1879), 70.

17 M. Fischberg, ‘Stosunki zdrowotne napływowej ludności żydowskiej w New-Jorku’, *Izraelita*, 43 (30 October 1903), 551. Cf. also: ‘“Sine ira et studio”. Słowo o Żydach ich kwestii’, *Izraelita*, 1 (4 January 1889), 6.

18 ‘Małżeństwa między krewnymi’, *Izraelita*, 4 (24 January 1908), 36.

19 E.g., ‘Indifferentyzm religijny’, *Izraelita*, 6 (25 May 1866), 41; ‘Chasydyzm, jego istota i stosunek do rabinizmu’, *Izraelita*, 27 (10 July 1868), 218; ‘Ocalenie. Powieść historyczna z początku 18-go wieku L. Filipsohna – przekład S.’, *Izraelita*, 32 (13 August 1869), 275. For more on this topic, see: Piotr Smoliński and Jakub Zygmunt, ‘Language as an Instrument of Marginalization. Discourse on People with Disabilities in the Trilingual Jewish Press Published in the Kingdom of Poland (1860s–1914)’ (forthcoming). See also: John M. Sefel, *Staging The [Disabled] Jew, op. cit.* (note 4), ii, 53, 80, 125 and *passim.*

20 ‘Fun arbe pines oylem’, *Haynt*, 144 (5 July 1912), 5.

21 ‘A kleyne bakoshe’, *Haynt,* 99 (12 May 1912), 5.

22 Some of them resemble ‘the disaster stories’ discussed by Julia Watts Belser, ‘Disability and the Social Politics of “Natural” Disaster: Toward a Jewish Feminist Ethics of Disaster Tales’, *Worldviews* 19, 1 (2015), 51–68. Likewise, they were not ‘ethically neutral’ and were said to ‘emerge from and reinforce the particular political, social and theological framework,’ *idem*, 52.

23 See for instance, ‘Bióro infomacijne o nędzy wyjątkowej’, *Izraelita*, 13 (25 March 1870), 104–5; ‘Bióro infomacijne o nędzy wyjątkowej’, *Izraelita*, 18 (6 May 1870), 148. Such announcements— including tables of names verified by the Office for Extreme Hardship (Biuro Nędzy Wyjątkowej)—were an initiative of the largest charitable organisation in the capital city, the Warsaw Charitable Society. These lists were not limited to any religious denomination, as they included both Christians and Jews, although with a greater number of Christians than the proportions of inhabitants would suggest.

24 ‘Zebranie głuchoniemych’, *Izraelita*, 9 (3 March 1911), 11; M. D., ‘A ferzamlung fun toyb-shtime’, *Haynt,* 38 (26 February 1913), 4–5.

25 ‘Der blinden-kongres in Broynshvayg’, *Haynt*, 166 (31 July 1912), 2.

26 ‘The general public is not very interested in the inner life or psychology of the blind, and a lot of people do not realise that many blind people can read and write and that they even have special libraries. The largest library for the blind is in France. It is the library of the Society named after Valentin Haüy, inventor of the convex alphabet, which blind people touch with their fingers. This library contains 9,000 volumes and spiritually satisfies nearly 40,000 blind people in France. The library building itself is extremely interesting. It has no windows and is completely dark, as no one there needs light, for even the librarians and their assistants are blind. In places there are convex stuccos [Yid. *shtukaturkes*] on the walls, so that blind people – in case they get lost – can find the right way by touching the walls. There is a postal department at the library, which sends books to blind people living in the province.’ ‘Fun arbe pines oylem’, *Haynt*, 5 (18 January 1912), 2. See also: ‘Bikher far blinde’, *Der Moment*, 22 (8 February 1911), 2.

27 ‘Ogłoszenie’, *Nowa Gazeta*, 377 (18 August 1912), 6.

28 Most likely to create an even more professional impression, the author of the advertisement referred interested individuals to the Warsaw Medical Society for more information, instead of directly to himself (although he also provided his address). In this way, he legitimised his position as a well-known member of the medical community.

29 ‘Ogłoszenie’, *Nowa Gazeta,* 315 (13 July 1911), 7.

30 ‘The director of the Łódź School for the Deaf welcomes 2 deaf children to his private apartment for complete maintenance. Teaching is according to the latest system. Special attention is paid to learning pronunciation. Truly parental care is diligently provided. Information: Łódź, 11 Nowocegielniana Street. I[zrael] Lichtenstein. In person: from 3 to 5 in the afternoon.’ *Nowa Gazeta*, 504 (3 November 1911), 9.

31 ‘Di oyg’, *Der Moment*, 232 (22 October 1913), 6. Idiomatic translation by: Anthony Goltz.

32 See for instance: Carmen M. Mangion , ‘“The Business of Life”: Educating Catholic Deaf Children in Late Nineteenth-Century England’, History of Education, 41, 5 (2012), 575–594 ; Iain Hutchison , ‘Oralism: A Sign of the Times? The Contest for Deaf Communication in Education Provision in Late Nineteenth-century Scotland’, European Review of History–Revue Européenne d’Histoire, 14, 4 (2007), 481–501 ; Ferdinand Berthier, *Forging Deaf Education in Nineteenth-century France: Biographical Sketches of Bébian, Sicard, Massieu, and Clerc,* ed. and transl.: Freeman G. Henry (Washington, D.C.: Gallaudet University Press, 2009) [translation of a nineteenth-century study]. The most rapid growth concerned institutions in the United States. For an account of this development, see: R.A.R. Edwards , Words Made Flesh: Nineteenth-century Deaf Education and the Growth of Deaf Culture (New York: NYU Press, 2012).

33 Emblematic of this attitude was a rhyming piece found in *Der Moment*, *op. cit.* (note 31), as well as the following press quote: ‘The cultural development of recent times has brought about significant and important changes in various aspects of life, but the greatest benefit has come to the unfortunate people who were born blind. These unfortunate people, who were once a burden to society and a curse to their families, can now, thanks to the latest educational methods, become fully useful people and earn their bread honestly.’ ‘Der blinden-kongres in Broynshveyg‘, *Haynt*, 166 (31 July 1912), 2. A similar comment regarding the deaf: N. L., ‘Visenshaftlikher felyeton. Toybe zolen heren’, *Haynt*, 127 (15 June 1914), 3.

34 For a thorough analysis on the language used about Jews with disabilities based on this research project, see: Piotr Smoliński and Jakub Zygmunt, ‘Language as an Instrument of Marginalization’, *op. cit.* (note 19).

35 ‘Z miasta. Niesmaczna reklama’, *Nowa Gazeta*, 351 (3 August 1911), 3. Cf. the story of a positively portrayed man of short stature who served in the royal courts: N, ‘Der “karlik fun di kenigen”’, *Haynt*, 131 (22 June 1909), 2.

36 Natan M. Meir, *Stepchildren of the Shtetl, op. cit.* (note 3), 8–9.

37
*Ibid.*, 148.

38 ‘Nowy szpital warszawskiej gminy żydowskiej’, *Izraelita*, 24 (19 June 1891), 204.

39 ‘Yediyot ha-olam ve-ha-teva. Holey ha-guf ve-holey ha-nefesh’, *HaTsefira*, 28 (26 July 1876), 4–5; ‘Zichronot me-asfat ha-rofim be-Romi’, *HaTsefira*, 95 (11 May 1894), 3.

40 T., ‘Di meshugoim-frage in Varsha’, *Haynt,* 182 (20 August 1913), 5.

41 A-Sh., ‘”Zofyuvka”’, *Haynt*, 133 (23 June 1912), 5.

42 Wojciech Gasik, ‘Rozwój praktyki i teorii pedagogiki specjalnej w wieku XIX i w początkach XX wieku’, in Stanisław Mauersberg (ed.), *Dzieje szkolnictwa i pedagogiki specjalnej* (Warszawa: PWN, 1990), 86–94. An exception in the West was the Netherlands: Ido Weijers , ‘Educational Initiatives in Mental Retardation in Nineteenth-century Holland’, History of Education Quarterly, 40, 4 (2000), 460–76.

43
*Ibid.*, 460–1.

44 Pioneering were the works of Édouard Séguin: *Traitement Moral, Hygiène, et Education des Idiots* (Paris: Baillière, 1846) and *Idiocy: and its Treatment by the Physiological Method* (New York: W. Wood and Co., 1866). See also: Jan D. Georgens and Heinrich M. Deinhardt , *Die Heilpädagogik – Mit besonderer Berücksichtigung der Idiotie und der Idiotenanstalten* . Zwölf Vorträge zur Einleitung und Begründung einer Heilpädagogischen Gesammtwissenschaft, vols 1-2 (Leipzig: Fleischer Verlag, 1861–1863).

45 For more on the topic, see e.g., Anne Digby and David Wright (eds), From Idiocy to Mental Deficiency: Historical Perspectives on People with Learning Disabilities (London: Routledge, 2002); Mark Jackson , The Borderland of Imbecility: Medicine, Society and the Fabrication of the Feeble Mind in Late Victorian and Edwardian England (Manchester: Manchester University Press, 2000).

46 In 1904 Germany, there were almost 700 special classes for the ‘mentally retarded’ in over 130 towns, providing education for around 15,000 children, Wayne L. Sengstock et al., ‘Germany, Cradle of American Special Education for Persons who are Mentally Retarded’, Education and Training in Mental Retardation, 25, 1 (1990), 5–6 .

47

Władysław Ołtuszewski , Niedorozwój psychiczny (idjota, głuptak, zacofany): Istota, zapobieganie i leczenie (Warszawa: M. Arct, 1905); earlier only his articles were published: ‘Zadania rodziców oraz społeczeństwa względem dzieci umysłowo upośledzonych (z niedorozwojem psychicznym)’, *Przegląd Pedagogiczny*, 21 (1899), 189–91; *ibid.*, 22 (1899), 198–9; *ibid.*, 23 (1899), 211–3. See also Wojciech Gasik, ‘Rozwój praktyki i teorii…’, *op. cit.* (note 42), 94–5, 101.

48 Wojciech Gasik, ‘Rozwój praktyki i teorii…’, *ibid.*, 102; Tomasz Fetzki, ‘Mniej znane oblicze “Starego Doktora”’, *Biografistyka Pedagogiczna* 6, 1 (2021), 97–100. Lublinerowa was active in the public sphere. For instance, in 1910 she participated in the International Congress on School Hygiene in Paris and afterwards gave a lecture in Warsaw ‘on the issue of abnormal children’ at the congress. Indicative of her position might have been the fact that two years later the commission of the Polish Society for the Study of Children (Polskie Towarzystwo Badań nad Dziećmi) held an official meeting in her apartment, *Pamiętnik Polskiego T-wa badań nad dziećmi za rok 1910* (Warszawa: Czcionkami Drukarni Naukowej, 1910), 8; ‘Doniesienia’, *Nowa Gazeta*, 8 (5 January 1912), 3. Her publications include: ‘Szkoła dla dzieci niedorozwiniętych, jej zadania, środki i rezultaty’, *Zdrowie* (1910), 633–41; ‘Teraźniejszość, przeszłość i przyszłość niedorozwiniętego dziecka’, *Głos Nauczycielski: organ Zrzeszenia Nauczycielstwa Polskich Szkół Początkowych*, 1, 10 (10 June 1918), 455.

49 B. G. ‘Instytut wychowawczo-naukowy dla dzieci niedorozwiniętych’, *Izraelita*, 50 (20 December 1907), 537; Arjel, ‘Dzieci upośledzone’, *Izraelita,* 44 (6 November 1908), 430; ‘Zakład dla umysłowo upośledzonych’, *Ziemia Lubelska*, 82 (23 March 1908), 3; ‘Szkoła dla mało rozwiniętych dzieci’, *Życie Illustrowane: dodatek do Kurjera Litewskiego*, 2/50 (27 December 1908), 406–7; see also: Tomasz Fetzki, ‘Mniej znane oblicze…,’ *op. cit.* (note 48), 100.

50

David Assaf (ed.), Journey to a Nineteenth-Century Shtetl: The Memoirs of Yekhezkel Kotik (Detroit: Wayne State University Press, 2008), 186 .

51 ‘Z kaznodziejskich dykteryj’, *Izraelita,* 1 (2 January 1891), 8. Similarly, in Mendele Moycher Sforim’s novel *Fishke der krumer* (*Fishke the Lame*), the protagonist is forced to marry a blind orphan when her first groom fails to show up at the wedding. Later, when the woman begins an affair with a rogue, Fishke falls in love with a hunchbacked girl, Natan M. Meir, *Stepchildren of the Shtetl, op. cit.* (note 3), 77.

52 Untitled, *HaTsofe*, 76 (10 April 1903), 8. A similar approach is found in the following passage from a literary text published in *HaTsofe*: ‘When an old matchmaker [Hebr. *shadkhan*] came to him and proposed a match [Hebr. *shidukh*], he thought to himself that the bride-to-be was most likely disabled. Otherwise, why would she choose him as her husband?’, M. Ben Eliezer, ‘HaRaav’ [Hunger], *HaTsofe,* 343 (3 March 1904), 2. Cf. also the 1892 novel by British-Jewish author Israel Zangwill, recalling a father who forbade his son to marry ‘a cripple’, i.e., a girl with a physical defect of the leg. The protagonist mentioned his ‘father’s fastidious objection to a dislocated bone’ and his insistence: ‘No, I want the straight–legged sister.’ Israel Zangwill, *Dreamers of the Ghetto* (Read Books Ltd, 2019).

53 Natan M. Meir, *Stepchildren of the Shtetl, op. cit.* (note 3), 9–10.

54 ‘O instytucjach gminy starozakonnych w Warszawie’, *Izraelita,* 6 (26 May 1866), 45–6; ‘W sprawie przytułków dla obłąkanych Żydów’, *Izraelita,* 46 (1 December 1899), 510–1. For an account explicitly describing a blind individual and a cripple as ‘useless’, see: ‘Przykłady filozofii rabinicznej’, *Izraelita*, 32 (13 August 1869), 275; a similar approach: ‘Der blinden-kongres in Broynshvayg’, *Haynt*, 166 (31 July 1912), 2.

55 Natan M. Meir, *Stepchildren of the Shtetl*, *op. cit.* (note 3), 3.

56 For a detailed commentary on the functioning of a *hekdesh*, see: Natan M. Meir, *Stepchildren of the Shtetl, op. cit.* (note 3), 28–32, 63–88.

57 ‘Fun ale eken shtodt’, *Haynt*, 31 (18 February 1912), 5. See also: Aneta Bołdyrew, ‘Udział lekarzy pochodzenia żydowskiego w popularyzacji wiedzy i działalności społecznej w Królestwie Polskim na przełomie XIX i XX w.’, *Studia Gdańskie*, 41 (2017), 36. Such generic charities were typical for the century, in contrast to later single-disease institutions (see e.g.: Malcolm Nicolson and George W. Lowis , ‘The Early History of the Multiple Sclerosis Society of Great Britain and Northern Ireland: A Socio-Historical Study of Lay/Practitioner Interaction in the Context of a Medical Charity’, Medical History, 46, 2 (2002), 141–74)12024806 PMC1044493.

58 See e.g.: ‘Obraz dobroczynności gminy starozakonnych w Warszawie przez H. Nussbauma’, *Jutrzenka*, 3 (19 July 1861), 19; ‘Kronika’, *Izraelita*, 44 (13 November 1891), 420.

59 Not all public assistance was acceptable. One of the issues often discussed in the press was begging, which was seen as an unacceptable way of receiving assistance. Pretending to be disabled when begging (in order to avoid work) was penalised—those caught were to be arrested. The only group exempted from the ban were those who had permission from religious authorities to beg in front of a church or cemetery. ‘Żebranina na ulicach’, *Nowa Gazeta*, 447 (30 September 1911), 2.

60 ‘A patient is brought in, a glance is taken at his nose, and he is told: “No vacancies!” A sick person comes to the infirmary, a crooked look is taken at his coat, and he is told: “No time! The doctor is busy today!”’, Noyekh Prilutski, ‘Der shreklikher rikshrit’, *Der Moment*, 23 (9 February 1913), 2.

61 The situation in the Saint John of God hospital in Warsaw was significantly worse: it hosted 400 patients having a capacity for only 250; the Jewish press deplored that ‘60 patients sleep in corridors without beds. ‘Z miasta’, *Nowa Gazeta,* 79 (18 February 1911), 2.

62 ‘Obraz dobroczynności gminy starozakonnych w Warszawie przez H. Nussbauma’, *Jutrzenka*, 3 (19 July 1861), 19.

63 This attitude remained stable throughout the analysed period, see e.g.: ‘O żebractwie’, *Jutrzenka*, 12 (18 September 1861), 92–3; ‘O dobroczynności prywatnej’ *Jutrzenka*, 19 (8 November 1861), 153; ‘Obraz statystyczny Głównego Domu Schronienia Ubogich i Sierot Starozakonnych w Warszawie’, *Jutrzenka,* 40 (3 October 1862), 331; ‘Słowo w swoim czasie’, *Izraelita,* 7 (14 February 1868), 49; ‘Biedni bez opieki’, *Izraelita*, 45 (24 November 1876), 356; ‘Rodzina Miljonerów’, *Izraelita,* 45 (25 November 1881), 370; ‘Różne’, *Nowa Gazeta*, 226 (18 May 1912), 2. Cf. also articles in the Polish press: ‘Wiadomości bieżące’, *Kurjer Warszawski,* 190b (28 July 1883), 12; ‘Ważna potrzeba’, *Słowo*, 135 (17 June 1897), 1.

64 Throughout Europe, large institutions were set up to confine hundreds of mentally ill people, such as Lunatic Asylum of the City of Berlin at Dalldorf (Irrenanstalt der Stadt Berlin zu Dalldorf). In addition, legislation was passed to reinforce the policy of confinement. The 1838 Law on Lunatics (La loi sur les aliénés) in France and the 1845 Lunatics Act in England changed the status of the mentally ill to that of patients.

65 T., ‘Di meshugoim-frage in Varsha’, *Haynt*, 182 (20 August 1913), 5; see also: ‘Der psikhyatoren-tsuzamenfohr in Peterburg. Vifiel gefinen zikh geystig-kranke in Rusland?’, *Haynt*, 301 (12 January 1910) 2; Elyash, ‘Meshugoim’, *Der Moment*, 19 (9 December 1910), 4; ‘Kultura a choroby umysłowe’, *Izraelita,* 35 (16 December 1910), 3.

66 ‘Ferantvortlikhkeyt far hezeykes fun meshugoim’, *Haynt*, 165 (31 July 1913), 5. As noted by Zygmunt and Smoliński, ‘Language as an Instrument of Marginalization’, *op. cit.* (note 19), these articles often had a sensational character, as with this example on the Warsaw Jewish community, deploring the absence of an institution dedicated to people with mental illnesses: ‘The lunatics are kept by their families in cramped, dark rooms, where even small children are kept, so it is easy to imagine what happens in such a room: in addition to the harm the lunatics cause, the family cannot feel safe …. A year ago, or more, it was discovered on Miła Street that an insane girl strangled her own mother. And this is not an isolated case, similar incidents have probably occurred more than once, but who can remember all these terrible things?’, Elyash, ‘Meshugoim’, *Der Moment*, 19 (9 December 1910), 4.

67 ‘Der entlofener fun Tvorki’, *Haynt*, 144 (5 July 1914), 5; ‘Der sof fun Dzhek der boykhshnayder’, *Haynt*, 175 (18 August 1908), 2; ‘Meshugoim in arest’, *Haynt*, 154 (18 July 1911), 3; ‘Fun gerikhts-zal: Obklangen fun onfal oyf Skalon’en’, *Haynt*, 13 (28 January 1912), 6; ‘Fun ale eken shtodt’, *Haynt*, 24 (10 February 1913), 4; ‘Telegramot. Mifnim ha-aretz’, *HaTsofe*, 196 (4 September 1903), 3; ‘Kronika’, *Nowa Gazeta,* 363 (9 August 1912), 1. Cf. also the remarks of Natan M. Meir, *Stepchildren of the Shtetl*, *op. cit.* (note 3), 7, on the interconnectedness of crime and marginality.

68 The press pointed to the principal reason for detention: making noise at night, disturbing other inhabitants’ sleep. Elyash, ‘Meshugoim’, *Der Moment*, 19 (9 December 1910), 4.

69 ‘Ogłoszenie’, *Nowa Gazeta*, 243 (29 May 1912), 8.

70 ‘A section for the insane, intended to accommodate 80 patients, half male and half female, and for this purpose the building itself, as well as the garden spread behind it, *enclosed by a fence*, will be divided *into two equal parts, completely isolated* [emphasis added]…there will also be two fairly large protrusions, where staircases will be arranged, and rooms for madmen … and many details, taking into account the safety of those doubly unfortunate patients,’ ‘Z miasta i kraju’, *Izraelita*, 24 (19 June 1891), 204.

71 ‘Di idishe gemeynde in Varsha’, *Haynt*, 205 (22 September 1908), 3.

72 ‘Fun ale eken shtodt’, *Haynt*, 31 (18 February 1912), 5. On the idea of deservedness see also: Natan M. Meir, *Stepchildren of the Shtetl*, *op. cit.* (note 3), 19–20, 53–4.

73 In 1823, Juliusz Falkowski, the Institute’s first director, explicitly wrote of the possibility of non-Christian children participating in classes in *O początkach i postępie Instytutu Warszawskiego Głuchoniemych oraz niektóre jego urządzenia dla wiadomości osób, jakikolwiek związek z Instytutem mieć mogących* (Warszawa, 1823), 63

74 In the school year 1873—1874, 8 of 166 deaf children were Jewish, although none of the Jewish children were blind (‘Pogadanki’, *Izraelita,* 41 (23 October 1874), 330). In 1896—1897, 13 of 218 deaf pupils and 3 of 40 blind pupils were Jewish (‘Kronika’, *Izraelita,* 16 (27 April 1900), 192). Some came from other cities, such as Abram Finkelstein from Radom in 1875, as for most of the century there was no other similar institution in the Kingdom (Archiwum Państwowe w Radomiu [State Archives in Radom], collection: Rada Opieki Społecznej Guberni Radomskiej, call no. 1098, 7). For lists of students, including Jews, see for instance: ‘Sprawozdanie z części wychowawczo-naukowej Warszawskiego Instytutu Głuchoniemych i Ociemniałych za rok szkolny 1872/3’, *Pamiętnik Warszawskiego Instytutu Głuchoniemych i Ociemniałych* (1873), V–XI; ‘Sprawozdanie z części wychowawczo-naukowej Warszawskiego Instytutu Głuchoniemych i Ociemniałych za rok szkolny 1873/4’, *Pamiętnik Warszawskiego Instytutu Głuchoniemych i Ociemniałych* (1874), V–X.

75 Statistical lists provided by some Warsaw newspapers on the number of deaf-blind candidates awaiting scholarships to the Warsaw Institute, with encouragement for private individuals to create scholarships in their names at the Institute for the maintenance of their pupils, had the desired effect: ‘The citizens of Warsaw, Mr. Leon Luwenstein and Mr. Józef Oranowski declared their willingness to create such scholarships, the first for one blind person and the second for one deaf person. From among the candidates for the blind, Stanislaw Brustmann, son of a private officer from Racławice in Kielce Gubernia [governorate], was chosen: from among the deaf-mute, Leonard Kaminski was selected, an orphan from Warsaw’, ‘Kronika’, *Izraelita*, 32 (14 August 1874), 259. See also: ‘Testament ś.p. Majera Bersohn’, *Izraelita*, 9 (28 February 1873), 70.

76 ‘Pogadanki’, *Izraelita*, 17 (2 May 1873), 137; ‘W kwestji dobroczynności gminnej’, *Izraelita*, 37 (19 September 1873), 295–6. For a list of contributors, including a considerable number of Jews, see: *Pamiętnik Warszawskiego Instytutu Głuchoniemych i Ociemniałych* (1873), 259–62. See also praise for numerous contributions from Lesser Levy in the official press of the Institute: ‘Sprawozdanie z obrotu funduszu głuchoniemych i ociemniałych’, *Pamiętnik Warszawskiego Instytutu Głuchoniemych i Ociemniałych* (1879), 356, 361.

77 ‘List do redaktora Izraelity o budowie synagogi w Warszawie przez Ben-Izaaka’, *Izraelita*, 45 (20 November 1874), 362.

78 One of the best-known examples is the activity of the British banker, activist, philanthropist and Sheriff of London, Sir Moses Montefiore. See: Abigail Green , Moses Montefiore. Jewish Liberator, Imperial Hero (Cambridge: Harvard University Press, 2010). Such activities were also mentioned in the Polish-Jewish press, e.g.: ‘Nowiny’, *Izraelita,* 16 (18 October 1867), 23; ‘Nowiny’, *Izraelita,* 23 (6 December 1867), 80.

79 Marcin Wodziński, ‘Legat Berka Sonnenberga czyli o zaskakującej karierze mimowolnego dobroczyńcy,’ *Studia Judaica,* 1, 13 (2004), 141–6.

80 ‘Wiadomości kraiowe’, *Dostrzegacz Nadwiślański,* 8 (25 February 1824), 59; Jan Papłoński, ‘Życiorysy dobroczyńców Instytutu’, *Pamiętnik Warszawskiego Instytutu Głuchoniemych i Ociemniałych* (1873), 108–11; ‘Światła i cienie’, *Izraelita,* 50 (25 December 1885), 402.

81 ‘Pogadanki’, *Izraelita*, 17 (2 May 1873), 137; ‘Sprawozdanie z części wychowawczo-naukowej Warszawskiego Instytutu Głuchoniemych i Ociemniałych za rok szkolny 1873/4’, *Pamiętnik Warszawskiego Instytutu Głuchoniemych i Ociemniałych* (1873), V–VI, XXIX. In July 1873, the press reported that, thanks to scholarships (*stypendia skarbowe*), among eighteen new deaf students, three were Jews, including one girl, all from Warsaw: Moses Hajdenwur/Mojżesz Handenwurcel (son of a tailor), Berek Kernbaum (son of a brazier), and Sura Szternlicht (daughter of a retired soldier), ‘Ogłoszenia’, *Izraelita*, 27 (11 July 1873), 220. It is assumed that the expression ‘stypendia skarbowe’ was synonymous with a scholarship based on Institute funds, largely using public finance.

82 Mojżesz Handenwurcel, mentioned above, received such an award after his first year. From her surname, we can assume that third-grade prize-winner Hanna Berngold was also Jewish. As for ‘commendation’ (differentiated from an ‘award’), this was directed to second grader Mojżesz Brańszczyk. ‘Sprawozdanie z części wychowawczo-naukowej Warszawskiego Instytutu Głuchoniemych i Ociemniałych za rok szkolny 1873/4’, *Pamiętnik Warszawskiego Instytutu Głuchoniemych i Ociemniałych* (1873), XXXIX–XL, XLII.

83
*Izraelita,* 1 (3 January 1873), 5-7; ‘Pogadanki’, *Izraelita,* 41 (23 October 1874), 330. For further explicit proof of his openness towards non-Catholics, see: Jan Papłoński, ‘Historyja Warszawskiego Instytutu Głuchoniemych i Ociemniałych’, *Pamiętnik Warszawskiego Instytutu Głuchoniemych i Ociemniałych* (1873), 55.

84 Indirect evidence of the Society’s denominational openness was the financial commitment of the above-mentioned Lesser Levy, vice president of the Jewish community board, who donated a far greater amount than most other donors to the organisation’s endowment fund, *Pięćdziesięciolecie Chrześcijańskiego Towarzystwa Głuchoniemych ‘Opatrzność’ w Warszawie* (Warszawa 1934), 12.

85 For more on these associations and the tavern, see: *Ibid.*, 5–8, 16; ‘Instytut głuchoniemych i ociemniałych w Warszawie’, in *Wielka Encyklopedia Powszechna Illustrowana*, 31–32 (Warszawa 1903), 18–9; Jacek Kulbaka, ‘Tradycje kształcenia specjalnego w Królestwie Polskim’, in Hanna Markiewiczowa and Iwona Czarnecka (eds), *Szkolnictwo, opieka i wychowanie w Królestwie Polskim. Od jego ustanowienia do odzyskania przez Polskę niepodległości 18151918* (Warszawa: Wydawnictwo Akademii Pedagogiki Specjalnej, 2016), 206; Bogdan Szczepankowski , Zarys historii stowarzyszeń głuchoniemych 1876–1946 (Warszawa: Zarząd Główny Polskiego Związku Głuchych, 1996), 19 ; Andrzej Kierzek , ‘Instytut Głuchoniemych i Ociemniałych w Warszawie w latach siedemdziesiątych XIX stulecia’, Otorynolaryngologia, 14, 2 (2015), 75–6.

86 ‘Pogadanki’, *Izraelita*, 26 (4 July 1879), 215. See also: ‘Światła i cienie,’ *Izraelita*, 27 (13 July 1883), 218. The social and cultural program of *Izraelita* emphasised Polish–Jewish co-operation, so correspondents were likely to focus on positive relations and overlook negative ones.

87 Statistics from 1900. *Pięćdziesięciolecie Chrześcijańskiego Towarzystwa*, *op. cit.* (note 84), 14; Jacek Kulbaka, *Tradycje kształcenia, op. cit.* (note 85), 201–2; Archiwum Państwowe w Warszawie [State Archives in Warsaw], collection: Instytut Głuchoniemych i Ociemniałych, call no. 1133, 3.

88 ‘Instytut głuchoniemych i ociemniałych w Warszawie’, in *Wielka Encyklopedia Powszechna*, *op. cit.* (note 85), 19.

89

Agnieszka Jagodzińska , Pomiędzy. Akulturacja Żydów Warszawy w drugiej połowie XIX wieku (Wrocław: Wydawnictwo Uniwersytetu Wrocławskiego, 2008), 75–8.

90 ‘Zebranie głuchoniemych’, *Izraelita,* 9 (3 March 1911), 11.

91 Archiwum Państwowe w Warszawie, collection: Instytut Głuchoniemych i Ociemniałych, call no. 1110, 13–4 (*Otchyot o sostoyanii Varshavskogo instituta glukhonemykh i slepykh v 1907 godu,* Varshava 1909 (*Ustav Varshavskago Instituta Glukhonemykh i Slepykh*, 1904).

92 An article from 1911 reported: ‘the lack of such a school can be felt all the more that in our country’s only existing such school – the Warsaw Institute for the Deaf-Mute – a percentage limit is in force. An important obstacle in this regard, however, is the lack of funds.’ ‘Zebranie głuchoniemych’, *Izraelita,* 9 (3 March 1911), 11. After a few months, the project was again discussed; its author was Mr Grützhandler, ‘Kronika’, *Nowa Gazeta,* 81 (19 February 1912), 3. As Tomasz Świderski has found, a project to set up a separate school was conceived already in 1901 and finally realised in 1923. Tomasz A. Świderski, *Przewodnik po Głuchej Warszawie* (Warszawa: Instytut Historii Głuchych ‘Surdus Historicus’, 2017), 36. From another press release, we learn about a 1911 ‘concert, combined with a dance party’, that was to be ‘held to benefit the construction of a shelter [Pol. *schronisko*] for blind and deaf Jewish children’, *Nowa Gazeta,* 456 (5 October 1911), 3. We do not, however, know if the term ‘shelter’ was, in this case, synonymous with the school (a board school) or whether it referred to another initiative.

93 M. D., ‘A ferzamlung fun toyb-shtime’, *Haynt*, 38 (26 February 1913), 4–5.

94 In the early 1900s, anti-Semitism and the idea of having to defend an ethno-national Polishness from the Jews became an integral part of the political discourse within the Kingdom of Poland. See for instance: Tadeusz Stegner , ‘Liberałowie Królestwa Polskiego wobec kwestii żydowskiej na początku XX wieku,’ Przegląd Historyczny, 80, 1 (1989), 69–88 .

95 M. D., ‘A ferzamlung fun toyb-shtime’, *Haynt*, 38 (26 February 1913), 4–5. For lists of association members, see: *Pięćdziesięciolecie Chrześcijańskiego Towarzystwa, op. cit.* (note 84) (‘Alfabetyczny spis członków’). Dora Gutfeld (née Eisenhorn), who joined the association in 1914, may have benefited from her Jewish husband Samuel Gutfeld’s long-standing membership (since 1892). Tomasz Świderski, *Przewodnik po Głuchej Warszawie*, *op. cit.* (note 92), 33, 94.

96 ‘It was decided to elect a committee that would take care of drafting and legalising the law of the Mutual Aid Society of Deaf Jews, at which there would also be a social club. Appointed to the executive committee were: L. Halpern, B. Krasucki, J. Weitzblum, and S. Gutfeld. ‘Zebranie głuchoniemych’, *Izraelita*, 9 (3 March 1911), 11. See also: M. D., ‘A ferzamlung fun toyb-shtime’, *Haynt*, 38 (26 February 1913), 4.

97 It was set up under the name: ‘Cohesion’: Association of Deaf-Mute Jews (Stowarzyszenie Głuchoniemych Żydów ‘Spójnia’). It was this body that organised the above-mentioned Jewish school for deaf children seven years later. Tomasz Świderski, *Przewodnik po Głuchej Warszawie*, *op. cit.* (note 92), 33, 36.

98 Archiwum Miasta Łodzi [State Archives in Łódź], collection: Policmajster w Łodzi, call no. 1883, 3–5, 7, 15.

99
*Ibid.*, 2–21.

100

Michał Trębacz , ‘Z dziejów żydowskiej edukacji w Łodzi: Izrael Lichtenstein - nauczyciel, pedagog, bundysta,’ Kronika Miasta Łodzi, 4 (2011), 61–69 ; Michał Trębacz, *Izrael Lichtenstein. Biografia żydowskiego socjalisty* (Łódź: Biblioteka Oddziału Instytutu Pamięci Narodowej w Łodzi, 2016). This school, largely forgotten by historiography, experienced a rapid increase in the number of students (in 1911 it had twenty-four pupils). In the Jewish press it was labelled as ‘the first school in the Kingdom of Poland, in which there are two departments with a two-class course of elementary schools,’ ‘Z Łodzi’, *Nowa Gazeta*, 102 (3 March 1911), 5; ‘Lodzer najes’, *Haynt*, 63 (28 March 1911), 3.

101 For example, in the Habsburg Empire a separate school for deaf Jewish children (Das Allgemeine Österreichische Israelitische Taubstummen-Institut) was established already in 1844 in the town of Nikolsburg (today Mikulov, in the Czech Republic); in 1852 it moved to Vienna. Another such institution operated in Lemberg (today Lviv, in Ukraine) from 1871. See: [Unknown], *Skizze über die allgemeine österr. israelitische Taubstummenanstalt in Wien* (Wien: Engel und Sohn, 1862), 3; Mirosław Łapot , ‘Działalność szkół i zakładów dla dzieci niemych i niewidomych, założonych z inicjatywy Żydów lwowskich w latach 1871–1939,’ Szkoła Specjalna, 4, (2018), 260–1.

102 Examples of such institutions: Aneta Bołdyrew, ‘Organizacja społecznej pomocy medycznej w Królestwie Polskim na przełomie XIX i XX w.’, *Piotrkowskie Zeszyty Historyczne*, 4 (2021), 49–82; Aneta Bołdyrew, ‘Źródła do badań nad ochronkami jako instytucjami opiekuńczo-wychowawczymi w Królestwie Polskim w XIX i na początku XX wieku’, *Studia Paedagogica Ignatiana*, 2 (2016), 271–89.

103

Judith Z. Abrams , Judaism and Disability: Portrayals in Ancient Texts from the Tanach through the Bavli (Washington DC: Gallaudet University Press, 1998), 87–99 . As Abrams puts it, Jewish religious teaching held that the ‘consequences of sin committed through the body are experienced through the body’ (*Ibid.*, 95). Among recent scholarship, noteworthy are the works of Julia Watts Belser, demonstrating the diversity of attitudes to disability within Judaism: Julia Watts Belser, ‘Priestly Aesthetics: Disability and Bodily Difference in Leviticus 21’, *Interpretation: A Journal of Bible and Theology*, 73, 4 (2019), 355–66; Julia Watts Belser, *Rabbinic Tales of Destruction: Gender, Sex, and Disability in the Ruins of Jerusalem* (New York: Oxford University Press, 2018); Julia Watts Belser, ‘Judaism and disability,’ in Darla Y. Schumm and Michael Stoltzfus (eds), *Disability and World Religions: An Introduction* (Waco: Baylor University Press, 2016), 93–113; Julia Watts Belser, ‘Brides and Blemishes: Queering Women’s Disability in Rabbinic Marriage Law’, *Journal of the American Academy of Religion*, 82 (2016), 401–29; Julia Watts Belser, ‘Reading Talmudic bodies: disability, narrative, and the gaze in Rabbinic Judaism,’ in Darla Schumm, Michael Stoltzfus (eds), *Disability in Judaism, Christianity, and Islam* (New York: Palgrave Macmillan, 2011), 5–27. Other important studies are: Bonnie L. Gracer , ‘What the rabbis heard: deafness in the Mishnah,’ in Darla Schumm , Michael Stoltzfus (eds), Disability in Judaism, Christianity, and Islam (New York: Palgrave Macmillan, 2011), 85–99 ; Ellen C. Wertlieb , ‘Attitudes towards Disabilities as Found in the Talmud,’ Psychology and Judaism, 12 (1988), 192–214 . More generally on Judaism and body: Mira Balberg , Purity, Body, and Self in Early Rabbinic Literature (Berkeley: University of California Press, 2014).

104 John M. Sefel, *Staging The [Disabled] Jew*, *op. cit.* (note 4), 84–86.

105 On this, see Marek Tuszewicki’s valuable study: A Frog Under the Tongue: Jewish Folk Medicine in Eastern Europe, transl. Jessica Taylor-Kucia (London: Littman Library of Jewish Civilization, 2021).

106 Henryk Lew, ‘O lecznictwie i przesądach leczniczych ludu żydowskiego’, *Izraelita*, 38 (24 September 1897), 363.

107 Henryk Lew, ‘O lecznictwie i przesądach leczniczych ludu żydowskiego’, *Izraelita*, 47 (3 December 1897), 456.

108
*Ibid.*, 456.

109
*Ibid.*, 456.

110 See: Marian Udziela, *Medycyna i przesądy lecznicze ludu polskiego* (Warszawa: Skład główny w księgarni M. Arcta, 1891).

111 Excellent comments on cholera weddings: Natan M. Meir, *Stepchildren of the Shtetl*, *op. cit.* (note 3), 13, 89–116; John M. Sefel, *Staging The [Disabled] Jew*, *op. cit.* (note 4), 98–119. See also: Hanna Węgrzynek , ‘Shvartze khasene. Black weddings among Polish Jews,’ in Glenn Dynner (ed.), Holy Dissent: Jewish and Christian Mystics in Eastern Europe (Detroit: Wayne State University Press, 2011), 55–68 .

112 Natan M. Meir, *Stepchildren of the Shtetl*, *op. cit.* (note 3), 89.

113 See for instance: Regina Lilientalowa, ‘Wierzenia, przesądy i praktyki ludu żydowskiego’, *Wisła,* 2 (1905), 76: ‘To remove the calamity during a major epidemic, such as cholera, equip the poorest couples, cripples, by marrying them in the cemetery.’

114 Natan M. Meir, *Stepchildren of the Shtetl*, *op. cit.* (note 3), 13.

115 ‘Czarne wesele’, *Słowo*, 176 (2 August 1894), 2.

116 ‘Za tydzień’, *Izraelita*, 42 (30 October 1891), 363.

117 ‘Z miasta i kraju’, *Izraelita*, 36 (11 September 1891), 306.

118 ‘Varshever tog-nayes. A khasune fun meshugoyim’, *Der Moment*, 7 (25 November 1910), 2.

119 ‘Fun der judisher gas in Varshe’, *Der Moment*, 19 (9 December 1910), 4. These references to past customs may be perceived to be a result of the lack of systematic knowledge about the aetiology of medical illnesses. In this context, it is worth mentioning Allen Thiher’s observation in *Revels in Madness: Insanity in Medicine and Literature* (Ann Arbor: University of Michigan Press, 2004), 163, 316, which describes how, in the nineteenth century, ‘medical theory about madness has really made no radical, disciplinary break with the past, such as occurred in medicine dealing with organic diseases’. This lack of knowledge might have encouraged the use of traditional concepts and solutions.

120 For excellent analyses of how disability is used within this novel, see: John M. Sefel, *Staging The [Disabled] Jew*, *op. cit.* (note 4), 88–99; Natan M. Meir, *Stepchildren of the Shtetl*, *op. cit.* (note 3), 77–85.

121 More broadly on literary disability studies, see for instance: Alice Hall , Literature and Disability (London-New York: Routledge Taylor and Francis Group, 2016), Alice Hall , The Routledge Companion to Literature and Disability (London-New York: Routledge Taylor and Francis Group, 2020); Ria Cheyne , Disability , Literature, Genre: Representation and Affect in Contemporary Fiction (Liverpool: Liverpool University Press, 2019); Joshua Essaka , Physical Disability in British Romantic Literature (Cambridge: Cambridge University Press, 2020); Michael Bérubé , ‘Disability and Narrative’, PMLA: Publications of the Modern Language Association of America, 120, 2 (2005), 568–76; Elisabeth A. Gitter , ‘Deaf-Mutes and Heroines in the Victorian Era’, Victorian Literature and Culture, 20 (1992), 179–96.

122

Lennard J. Davis , ‘Constructing normalcy: The bell curve, the novel, and the invention of the disabled body in the nineteenth century’, in Lennard J. Davis (ed.), The Disability Studies Reader. Second Edition (New York: Routledge Taylor and Francis Group, 2007), 11 .

123 However, it is worth mentioning that, for example, *Haynt* advertised plays and published theatre reviews of plays in which the main characters were people with disabilities. It is likely that making the title character a person with a disability was intended to provide an additional, emotionally stirring and intriguing attraction. See advertisements and reviews in *Haynt*, e.g.: ‘Der meshugener in shpitol’ [A Madman in the Hospital’], *Haynt*, 83 (25 April 1909), 4; ‘Der blinder muzikant’ [The Blind Musician], *Haynt*, 25 (11 February 1910), 8; ‘Der meshugener kompozitor’ [The Crazy Composer’], *Haynt*, 220 (5 November 1911), 4; ‘Bunt in meshugoim-shpitol’ [Rebellion in the lunatic asylum’], *Haynt*, 9 (23 January 1914), 7; ‘”Blind oys liebe” fun Z. Libin’, *Haynt*, 241 (3 November 1912), 4.

124 Shalom Asch, ‘Matka’, *Izraelita*, 22 (31 May 1912), 2.

125 Henryk Lichtenbaum, ‘W świecie zastoju’, *Izraelita*, 22 (22 September 1911), 6.

126 ‘Potwarca. Powieść historyczna’, *Izraelita*, 3 (16 January 1891), 25.

127 Yankev Mazl-Meyzel, ‘Sane der kapelmeyster. (A bildel)’, *Haynt*, 8 (22 January 1909), 3–4 and *ibid.*, 9 (24 January 1909), 2.

128 Natan S. Meir, *Stepchildren of the Shtetl*, *op. cit.* (note 3), 165.

129 Czesława Endelmanowa, ‘Nad trzęsawiskiem’, *Izraelita*, 5 (10 February 1905), 55. For another example of a literary portrayal of an individual characterised solely as a ‘hunchback’ see N. Samueli, ’Między młotem a kowadłem’, *Izraelita*, 25 (26 June 1891), 208.

130 Jan Adolf Hertz, ’Dzień przebaczenia’, *Izraelita*, 11 (17 March 1911), 4.

131 ‘Przygody głuchego weterana, opowiadanie dr-a L. Filipsona, spolszczyła S.’, *Izraelita*, 3 (23 January 1870), 22–3; *ibid.*, 1 (6 January 1871), 5; *ibid.*, 5 (3 February 1871), 37; *ibid.*, 6 (10 February 1871), 46; *ibid.*, 7 (17 February 1871), 55; *ibid.*, 9 (3 March 1871), 70; *ibid.*, 12 (24 March 1871), 93–5; *ibid.*, 3 (17 January 1873), 22–3.

132 K.E.F., ‘Przygody siłacza’, *Izraelita*, 7 (13 February 1891), 62.

133 One of the most prominent examples of multilingualism were two pioneers of Hebrew journalism: Hayyim Selig Słonimski, a mathematician, astronomer, inventor and founder of *HaTsefira*, and Nahum Sokołów, who wrote articles for *Izraelita* and *HaTsefira* and also served as their editor-in-chief.

134 Aneta Bołdyrew, ‘Udział lekarzy pochodzenia żydowskiego’, *op. cit.* (note 57), 28.

135 Teresa Ostrowska, ‘Lubliner Stanisław Leopold’, *Polski Słownik Biograficzny*, vol. XVII (Wrocław: Zakład Narodowy im. Ossolińskich, 1972); Aneta Bołdyrew, ‘Udział lekarzy pochodzenia żydowskiego’, *op. cit.* (note 57), 31.

136 Grzegorz Błaszczyk, *Dorpatczycy. Polscy studenci na Uniwersytecie Dorpackim w latach 1802–1918 i ich dalsze losy. Słownik biograficzny*, vol. II (Wydawnictwo Naukowe FNCE, 2023), 458–9; Tadeusz Zakrzewski, ‘Lubelski Wilhelm Szymon (1832–1891)’, *Polski Słownik Biograficzny*, vol. XVII (Wrocław: Zakład Narodowy im. Ossolińskich, 1972), 593–4. Biographical sketches of other famous Jewish doctors: Aneta Bołdyrew, ‘Udział lekarzy pochodzenia żydowskiego’, *op. cit.* (note 57), 27–40.

137

Douglas C. Baynton , Defectives in the Land: Disability and Immigration in the Age of Eugenics (Chicago: University of Chicago Press, 2016), 133 and *passim.* In this regard, the non-Jewish Polish press employed the term ‘pariah’. Evoking a ‘Dantean sphere of misery’, it deplored the condition of various groups of helpless people: ‘One such category of social pariah is the huge number of incapacitated, infirm old people, victims of incurable illness or disability [who] do not have the means for a tolerable existence.’ ‘Ważna potrzeba’, *Słowo*, 135 (17 June 1897), 1.

138 For details of an 1875 plan by the General Governor see: Archiwum Państwowe w Radomiu, collection: Rada Opieki Społecznej Guberni Radomskiej, call no. 1098, 2–4.

139 Articles on treating deafness were also published in the Jewish press; see for instance: N. L., ‘Visenshaftlikher felyeton. Toybe zolen heren’, *Haynt,* 127 (15 June 1914), 3. Countless works on the topic were published in the thriving medium of medical press: *Czasopismo Lekarskie* (1899–1908), *Gazeta Lekarska* (1866–1921), *Kronika Lekarska* (1879–1907), *Krytyka Lekarska* (1897–1907), *Medycyna* (1873–1907), *Pamiętnik Towarzystwa Lekarskiego Warszawskiego* (1837–1930), and *Przegląd chirurgiczny* (1893–1905); see, for example: ‘Wiadomości o nowym środku przeciwko głuchocie’, *Pamiętnik Towarzystwa Lekarskiego Warszawskiego*, vol. XLIII, S. III/I (1860), 448; ‘Kilkanaście przypadków leczonych na głuchotę, metodą Cléret’, *Pamiętnik Towarzystwa Lekarskiego Warszawskiego*, vol. XLIV, S. III/ II (1860), 121–3. From amidst numerous medical treaties on the aetiology and treatment of deafness, see e.g.: Karol Gregorowicz , O głuchocie chorobach sprowadzających głuchotę i sposobach leczenia głuchoty, szczególniej podług odkrycia Cleret siarczanym eterem (Warszawa: Skład główny w Księgarni Nowoleckiego, 1861). For more on the topic: Jan Wnęk , ‘Krytyka naukowa polskich książek medycznych na przełomie XIX i XX wieku’, Archiwum Historii i Filozofii Medycyny, 78 (2015), 76.

140 An increase in the number of people classified as mentally ill was a phenomenon observed throughout Europe. See e.g.: Pamela Dale and Joseph Melling , ‘The politics of mental welfare: fresh perspectives on the history of institutional care for the mentally ill and disabled’, in Pamela Dale and Joseph Melling (eds), Mental Illness and Learning Disability since 1850 (New York: Routledge, 2006), 1–23 .

141 John M. Sefel, *Staging The [Disabled] Jew, op. cit.* (note 4), 50; Shmuel Feiner , The Jewish Enlightenment (Philadelphia, PA: University of Pennsylvania Press, 2011), xii.

142 A part of this tendency was the general emphasis on hygiene typical of the wider European discourse of the time. John M. Sefel, *Staging The [Disabled] Jew, op. cit.* (note 4), 53, 63–4, 68.

143 Natan M. Meir, *Stepchildren of the Shtetl*, *op. cit.* (note 3), 5–6. On the concept of the ‘New Jew’, see for instance: Yitzhak Conforti , ‘“The New Jew” in the Zionist movement: ideology and historiography’, The Australian Journal of Jewish Studies, 25 (2011), 87–119 .

144 Natan M. Meir, *Stepchildren of the Shtetl*, *op. cit.* (note 3), 4.

145
*Ibid.*, 83.

146

Harlan Lane , The Mask of Benevolence. Disabling the Deaf Community (New York: Alfred A. Knopf, 1992).

147

Ashley Taylor , ‘The Discourse of Pathology: Reproducing the Able Mind through Bodies of Color,’ Hypatia, 30, 1 (2015), 182.

148

Dominika Czarnecka , ‘How do these *exotic* bodies move? Ethnographic shows and constructing otherness in the Polish-language press, 1880–1914,’ in Dagosław Demski and Dominika Czarnecka (eds), Staged Otherness: Ethnic Shows in Central and Eastern Europe, 1850–1939 (Budapest: Central European University Press, 2021), 201–28. For more on Jewish interest in another massively popular form of urban entertainment focused on the body, see Edward Portnoy , ‘Freaks, Geeks, and Strongmen: Warsaw Jews and Popular Performance, 1912–1930’, TDR: Drama Review, 50, 2 (2016), 117–35.

